# A novel framework to build saliva‐based DNA methylation biomarkers: Quantifying systemic chronic inflammation as a case study

**DOI:** 10.1111/acel.14444

**Published:** 2025-01-30

**Authors:** Lisa J. Schmunk, Toby P. Call, Daniel L. McCartney, Hira Javaid, Waylon J. Hastings, Vanja Jovicevic, Dragoljub Kojadinović, Natacha Tomkinson, Eliska Zlamalova, Kirsty C. McGee, Jack Sullivan, Archie Campbell, Andrew M. McIntosh, Veronika Óvári, Karl Wishart, Christian E. Behrens, Emma Stone, Miloš Gavrilov, Rob Thompson, Hurdle bio‐infrastructure team, Pierric Descamps, Emily Taylor, Chuck Paiusi, Leona Mc Girr, Ollie Philpott, Ian Robinson, Hector Watson, Ana Magalia, Dave Russell, David Woods, Thomas Jackson, Janet M. Lord, Thomas M. Stubbs, Riccardo E. Marioni, Daniel E. Martin‐Herranz

**Affiliations:** ^1^ Hurdle.Bio/Chronomics Ltd. London UK; ^2^ Centre for Genomic and Experimental Medicine, Institute of Genetics and Cancer University of Edinburgh Edinburgh UK; ^3^ Department of Psychiatry and Behavioral Sciences Tulane University School of Medicine New Orleans Louisiana USA; ^4^ MRC‐Versus Arthritis Centre for Musculoskeletal Ageing Research, Institute of Inflammation and Ageing University of Birmingham Birmingham UK; ^5^ Division of Psychiatry, Centre for Clinical Brain Sciences University of Edinburgh Edinburgh UK; ^6^ Bayer Consumer Care AG Basel Switzerland; ^7^ Bayer HealthCare LLC Whippany New Jersey USA; ^8^ NIHR Birmingham Biomedical Research Centre University Hospitals Birmingham Birmingham UK; ^9^ Present address: Pale Fire Capital SE Prague Czech Republic

**Keywords:** ageing, biomarker, DNA methylation, epigenetic clock, inflammageing, machine learning, systemic chronic inflammation (SCI)

## Abstract

Accessible and non‐invasive biomarkers that measure human ageing processes and the risk of developing age‐related disease are paramount in preventative healthcare. Here, we describe a novel framework to train saliva‐based DNA methylation (DNAm) biomarkers that are reproducible and biologically interpretable. By leveraging a reliability dataset with replicates across tissues, we demonstrate that it is possible to transfer knowledge from blood DNAm to saliva DNAm data using DNAm proxies of blood proteins (EpiScores). We apply these methods to create a new saliva‐based epigenetic clock (InflammAge) that quantifies systemic chronic inflammation (SCI) in humans. Using a large blood DNAm human cohort with linked electronic health records and over 18,000 individuals (Generation Scotland), we demonstrate that InflammAge significantly associates with all‐cause mortality, disease outcomes, lifestyle factors, and immunosenescence; in many cases outperforming the widely used SCI biomarker C‐reactive protein (CRP). We propose that our biomarker discovery framework and InflammAge will be useful to improve understanding of the molecular mechanisms underpinning human ageing and to assess the impact of gero‐protective interventions.

## Graphical abstract

In this study, we describe a novel multi‐omics framework to train saliva‐based DNA methylation (DNAm) biomarkers that are reproducible and biologically interpretable. By leveraging a reliability dataset with replicates across tissues, we demonstrate knowledge transfer from blood DNAm to saliva DNAm data using DNAm proxies of blood proteins (EpiScores). We apply these methods to create a new saliva‐based epigenetic clock (InflammAge) that quantifies systemic chronic inflammation (SCI) in humans.

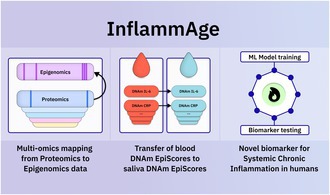

**Abbreviations**
AgeAccel: epigenetic age acceleration
ANOVA: one‐way analysis of variance
BEST: NIH‐FDA Biomarkers EndpointS and other Tools classification framework
BMI: body mass index
BMP1: Bone Morphogenetic Protein 1
CCL11: C‐C Motif Chemokine Ligand 11
CCL18: C‐C Motif Chemokine Ligand 18
CI: confidence interval
COPD: chronic obstructive pulmonary disease
COVID‐19: coronavirus disease 2019
CpG: 5'‐cytosine‐phosphate‐guanine‐3'
CRP: C‐reactive protein
CXCL10: C‐X‐C Motif Chemokine Ligand 10
CXCL9: C‐X‐C Motif Chemokine Ligand 9
DNA: Deoxyribonucleic acid
DNAm: DNA methylation
EHR: electronic health record
Eos: eosinophils
EpiScore: Epigenetic proxy score to predict analyte levels from DNAm data
ESR: erythrocyte sedimentation rate
EWAS: epigenome‐wide association study
FAP: Fibroblast Activation Protein Alpha
FDR: false discovery rate
GDF‐15: Growth Differentiation Factor 15
GEO: Gene Expression Omnibus
GO: Gene ontology
GS: Generation Scotland
hg19: Human genome version 19, equivalent to Homo sapiens (human) genome assembly GRCh37
HR: Hazard Ratio
iAge: InflammationAge (Sayed et al 2021) proxy of inflammageing based on protein and cytokine measurements
ICC: intraclass correlation coefficient
IgG: Immunoglobin G
IL‐18R1: Interleukin 18 receptor 1
IL‐6: Interleukin 6
InflammAge: Novel epigenetic biomarker to measure DNAm inflammation age (this work)
InflammAgeAccel: InflammAge acceleration
INF‐γ: Interferon‐gamma
MAE: median absolute error
MET: metabolic equivalents
ML: machine learning
MMP12: Matrix Metallopeptidase 12
Mono: monocytes
N: sample size
Neu: Neutrophils
NHL: Non‐Hodgkin's lymphoma
NK cell: Natural killer cell
NOTCH1: Notch Receptor 1
NS: non‐significant
NTRK3: Neurotrophic Receptor Tyrosine Kinase 3
PCA: principal component analysis
pQTL: genetic protein quantitative trait loci
Probe: Methylation array probe that hybridises with select genomic regions to determine CpG methylation status
*R*_*p*_
: Pearson correlation coefficient
*R*_*s*_
: Spearman correlation coefficient
SCI: systemic chronic inflammation
TNFRSF1B: TNF receptor superfamily member 1B
TNF‐α: tumour necrosis factor‐alpha
UTR: untranslated regions

## 1 INTRODUCTION

DNA methylation (DNAm) is a powerful data type to build biomarkers that quantify the risk for developing age‐related diseases and the ageing process itself (Horvath & Raj, 2018). This may be due to the ability of DNAm to capture information from both genetics and lifestyle/environmental factors. DNAm patterns associated with ageing and maximum lifespan are evolutionarily conserved (Lu et al., 2023), suggesting a critical role in the biology of ageing.

Epigenetic ageing clocks are becoming more widespread in clinical research, including in the context of quantifying the effect of candidate healthspan extending interventions in human trials (Moqri et al., 2023), and in the consumer market for preventative healthcare applications. This has been driven by significant improvements in the design and performance of these biomarkers, both from a technical and clinical perspective. Early examples include the multi‐tissue Horvath clock (Horvath, 2013), the blood‐specific Hannum clock (Hannum et al., 2013) or the skin‐blood clock (which has good predictive performance in saliva samples and in vitro cell culture) (Horvath et al., 2018). These were trained directly on chronological age and are able to predict it with remarkable accuracy, which has applications in the forensic field (Horvath & Raj, 2018).

Second‐generation epigenetic clocks have improved associations with clinical outcomes. Instead of training DNAm biomarkers directly on chronological age, it is possible to train them on intermediate phenotypic proxies that better capture morbidity and all‐cause mortality risk. These include phenotypic ageing measurements derived from clinical biomarkers (PhenoAge) (Levine et al., 2018), a proxy that quantifies the pace of ageing across multiple organs/systems (DunedinPACE) (Belsky et al., 2022) or DNAm‐based predictors of plasma proteins and smoking exposure that accurately predict all‐cause mortality (GrimAge) (Lu et al., 2019). Further methodological innovations, such as leveraging principal component analysis (PCA) to reduce technical variability of CpG probes, have enhanced the test–retest reliability of DNAm biomarkers, which is critical to capture longitudinal trajectories and intervention effects (Higgins‐Chen et al., 2022).

Despite these improvements, two problems remain for existing DNAm‐based biomarkers: (Aps et al., 2002) the lack of biological interpretability with current epigenetic clocks behaving like a “black box” (with the exception of perhaps GrimAge, where features can be interpreted), and (Aryee et al., 2014) the paucity of second‐generation reproducible epigenetic clocks in saliva (which is a more accessible and scalable sample type for preventative healthcare propositions when compared to blood). Here, we outline a novel framework to build saliva‐based DNAm biomarkers that addresses these challenges by leveraging a library of blood‐based DNAm predictors of different plasma proteins (protein epigenetic scores or EpiScores) (Aslibekyan et al., 2018; Gadd et al., 2022; Hillary et al., 2020; Ligthart et al., 2016; Stevenson et al., 2020, 2021).

Ageing is driven by many molecular processes known as “Hallmarks of Ageing” (López‐Otín et al., 2023), with the geroscience hypothesis postulating that measuring and targeting these processes rather than individual diseases will lead to a more successful impact on human healthspan (Kennedy et al., 2014). Low‐grade systemic chronic inflammation (SCI), also known as “inflammageing”, has recently emerged as one of these hallmarks (López‐Otín et al., 2023). SCI is characterised by persistent and non‐resolving inflammation in the absence of an acute trigger such as infection, leading to collateral tissue damage and an increase in disease risk (Furman et al., 2019).

A subset of blood‐based biomarkers traditionally associated with acute inflammatory responses also consistently change their baseline levels during human ageing; thus they have been proposed as SCI biomarkers. This includes biomarkers such as C‐reactive protein (CRP) (Tang et al., 2018), erythrocyte sedimentation rate (ESR) (Siemons et al., 2014), IL‐6 (Stevenson et al., 2021), TNF‐α (Koelman et al., 2019), interferon‐gamma (IFN‐γ) (Koelman et al., 2019), CXCL9 (Sayed et al., 2021), and other cyto‐ and chemokines (Koelman et al., 2019). However, due to the high biological variability of these biomarkers with acute inflammation and infection status, single measurements are normally insufficient to determine the SCI status of an individual.

Recently, omics‐based approaches have used machine learning (ML) algorithms to predict SCI status, including the immunoglobulin G (IgG) glycome (Štambuk et al., 2020) or iAge (Sayed et al., 2021). However, these biomarkers still require invasive procedures to access blood, they may not be very stable (e.g., cytokines in iAge) and they require access to expensive and complex molecular assays (which are not globally available across laboratories); all of which increase the barriers for widespread adoption in preventative healthcare settings.

In this work, we demonstrate the utility of our framework to train a new saliva‐based DNAm biomarker for SCI (hereafter termed InflammAge). InflammAge addresses the hurdles outlined above for SCI quantification while demonstrating high technical reliability, significant associations with clinical outcomes and increasing biological interpretability when compared to other epigenetic clocks.

## 2 RESULTS

### 2.1 A novel framework to train biologically‐interpretable and reproducible DNAm biomarkers in saliva

To harness the power of DNAm as a data type and saliva as an easily accessible human sample, we created a framework for developing novel saliva‐based DNAm biomarkers informed by biological interpretability.

Genome‐wide DNAm can be quantified using Illumina Infinium methylation microarrays. The most widely used technology is the Illumina EPIC array, which quantifies >850,000 CpG sites and was designed to enhance novel discovery. We aimed to create a more cost‐effective approach to DNAm assessment at sites relevant to geroscience. To generate this custom array, termed the Hurdle DNAm platform, we selected CpG sites with strong phenotypic associations established by published epigenome‐wide association studies (EWAS) (Battram et al., 2022; Li et al., 2019). We enriched for actionable traits, lifestyle and environmental exposures, epigenetic clocks and complex diseases (Tables S1 and S2); resulting in ~30,000 CpG sites that are good candidate features for biomarker development (see Section 4 and Figure S1). Genomic location analysis (see Section 4) revealed a higher proportion of probes in functional genomic sites (such as promoters, gene bodies, CpG islands and shores, and DNase Hypersensitivity Sites) in the Hurdle DNAm platform compared to all EPIC probes (Figure S2).

EpiScores are DNAm‐based predictors of varied traits with direct biological interpretability, such as blood protein levels (Gadd et al., 2022; Stevenson et al., 2020, 2021). As DNAm captures cellular states upstream of gene regulation, these EpiScores may act as de‐noised proxies for blood proteins, better capturing chronic disease risk. This may prevent confounding with short‐term fluctuations in blood concentrations, for example, due to circadian rhythms or acute infection. We propose that these EpiScores can be transferred from blood (where they were originally trained) to saliva; and then used as improved biological features to train ML models able to predict complex phenotypes from DNAm data (while still allowing for biological interpretability).

**FIGURE 1:**
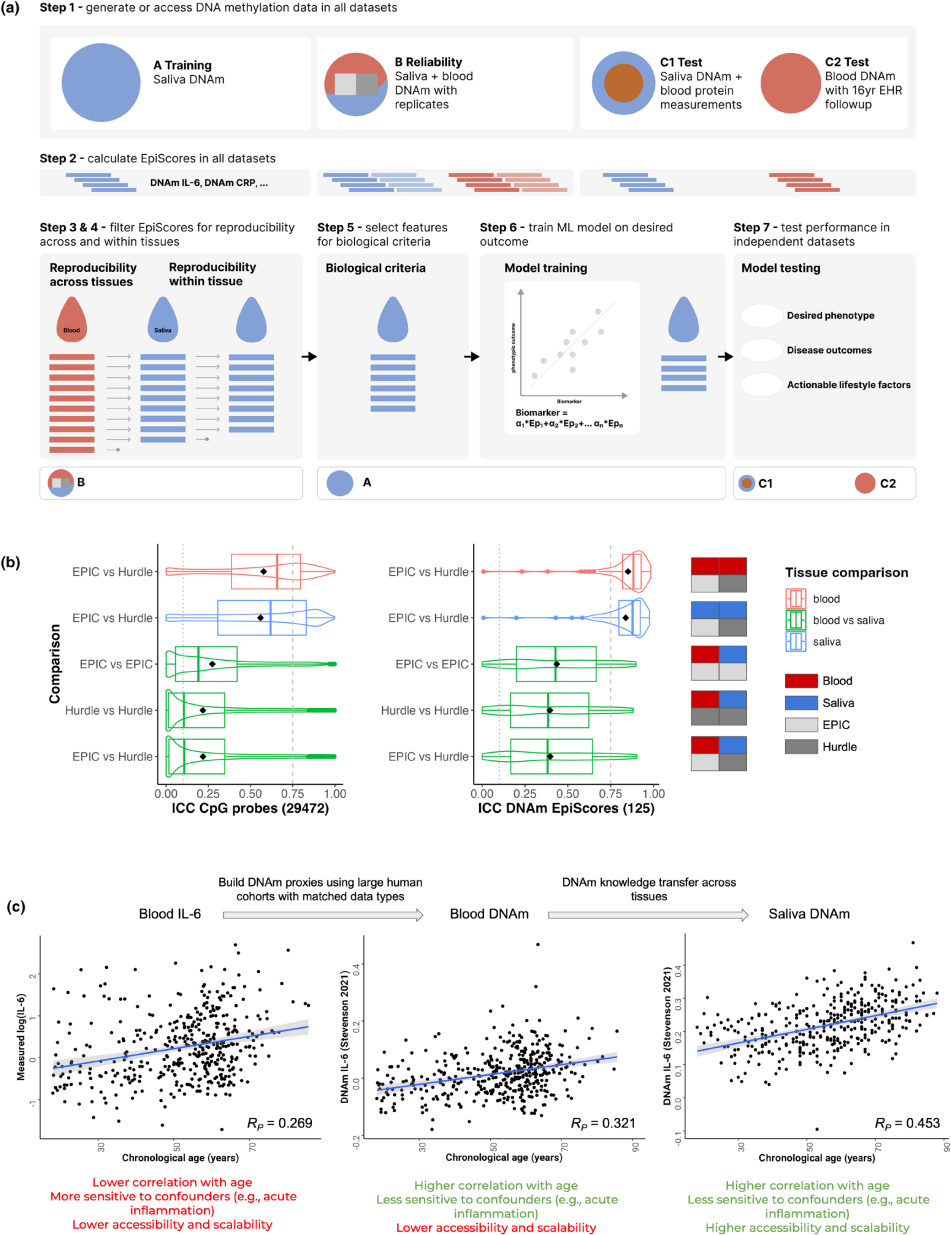
A novel framework to train biologically‐interpretable and reproducible DNAm biomarkers in saliva. (a) Framework steps (Aps et al., 2002; Aryee et al., 2014; Aslibekyan et al., 2018; Battram et al., 2022; Belsky et al., 2022; Bocklandt et al., 2011; Costantini et al., 2018) and datasets (training, reliability, test) required to train novel DNAm biomarkers in saliva. CRP, C‐reactive protein; EHR, electronic health records; IL‐6, Interleukin 6. (b) Reproducibility analysis for CpG probes (left) and EpiScore (right) features considered during training of InflammAge. Distributions of intraclass correlation coefficients (ICC) from the reliability dataset (V1 samples) are shown as box and violin plots for five comparisons (within/between blood and saliva); interquartile range = squares, mean = black diamond. Grey vertical lines indicate ICC filtering thresholds (0.75 for within and 0.10 between tissues). (c) Transfer of biological information from blood proteins (left, data: Stevenson et al., 2021), to blood DNA methylation EpiScores (center, data: Stevenson et al., 2021), to saliva DNAm EpiScores (right, data: Saliva training dataset). For InflammAge, saliva DNAm SCI features selected based on the association with ageing (chronological age) included the SCI biomarker IL‐6 which increases with age.

Our DNAm biomarker discovery framework consists of the following steps (Figure 1a):

1. generate genome‐wide DNA methylation data in all datasets;
2. calculate a library of published blood EpiScores as features in all datasets;
3. filter the calculated features in the training dataset by reproducibility across tissues using a reliability dataset with matched blood and saliva DNAm data;
4. filter the remaining features in the training dataset by technical reliability of each feature within the tissue (within blood/saliva), using technical replicates from a reliability dataset;
5. select final features in the training dataset based on biological criteria (e.g., association with physiological ageing, association with a disease process, etc);
6. train the final ML model on the desired phenotypic outcome (e.g., chronological age, all‐cause mortality, time‐to‐disease, etc) using the selected features in the training dataset in order to generate the DNAm biomarker; and
7. validate the final DNAm biomarker in independent test datasets.

Next, we describe how this hypothesis‐driven framework can be applied to build a novel saliva‐based DNAm biomarker of systemic chronic inflammation (SCI) in humans (InflammAge).

### 2.2 Applying the framework to train a saliva‐based DNAm biomarker for systemic chronic inflammation (InflammAge)

Different proteins (including cytokines) have been associated with SCI, providing evidence that this ageing hallmark can be quantified at the molecular level in blood. We observed the age‐related association of many of these biomarkers in one of our healthy ageing cohorts (see Alpha test dataset in Section 4), which included an increase with age in baseline levels of GDF‐15 (Spearman correlation coefficient *R*_*S*_ = 0.76, FDR‐adj. *p*‐value *p*_*FDR*_ = 3.8 × 10^−12^), CXCL9 (*R*_*S*_ = 0.67, *p*_*FDR*_ = 6.6 × 10^−9^), TNF‐α (*R*_*S*_ = 0.40, *p*_*FDR*_ = 4.9 × 10^−3^), IL‐6 (*R*_*S*_ = 0.36, *p*_*FDR*_ = 0.013), CRP (*R*_*S*_ = 0.33, *p*_*FDR*_ = 0.027), or CCL11 (*R*_*S*_ = 0.30, *p*_*FDR*_ = 0.0498) (Figure S3). Based on this, we collated a library of published blood DNAm CpG sites and EpiScores associated with cytokines and other inflammation‐related proteins. This includes CRP (Ligthart et al., 2016; Stevenson et al., 2020), IL‐6 (Stevenson et al., 2021), TNF‐α (Aslibekyan et al., 2018), CXCL9, CCL11, IL‐18R1 (Hillary et al., 2020), and 109 EpiScores containing inflammatory proteins measured in the Olink and SOMAscan platforms (Gadd et al., 2022). These features were calculated across all DNAm samples in all datasets (training, reliability, test; step 2 of the framework).

To ensure the reproducibility of these features, we generated a reliability dataset with DNAm technical replicates for matched saliva and blood samples by measuring methylation profiles for each tissue on both the EPIC array and the Hurdle DNAm platform. We calculated intraclass correlation coefficients (ICCs) between individual CpG probes present on both platforms (29,472) and for each summary EpiScore (125 in total) for the following comparisons: blood EPIC versus blood Hurdle, saliva EPIC versus saliva Hurdle, blood EPIC versus saliva EPIC, blood Hurdle versus saliva Hurdle, and blood EPIC versus saliva Hurdle (Figure 1b). Using this approach, we assessed which CpG probes and EpiScores could be measured reliably across platforms and tissues. We observed that EpiScores showed higher reliability both within tissues (mean values of ICC_Blood_ = 0.85, 95% confidence interval [0.76–0.90]; ICC_Saliva_ = 0.84 [0.74–0.90]) and between tissues measured on the same platform (mean ICC_Blood‐vs‐Saliva_ = 0.42 [0.13–0.61]) compared to means of individual CpG probes (ICC_Blood_ = 0.58 [0.42–0.69]; ICC_Saliva_ = 0.56 [0.40–0.68]; ICC_Blood‐vs‐Saliva_ = 0.25 [0.03–0.42]). Furthermore, many EpiScores showed strong positive correlations between blood EPIC and saliva Hurdle replicates collected from the same individual at the same time (Figure S4a), adding robust evidence to the ability to transfer EpiScores information from standard blood DNAm data to saliva DNAm data generated using the novel Hurdle platform. We therefore decided to train InflammAge using the EpiScores instead of the individual CpGs that comprise the EpiScores.

Using the V1 samples from the reliability dataset (see Appendix S1), different ICC thresholds for the EpiScores were benchmarked to optimise the ICC for the final InflammAge biomarker (Figure S5). This resulted in selecting EpiScores with ICC >0.10 between blood and saliva (step 3 of the framework; 101/125 EpiScores remained) and an ICC >0.75 within saliva and within blood replicates (step 4 of the framework; 86/125 EpiScores remained). The 86 EpiScores with high reliability were used downstream for training.

**FIGURE 2:**
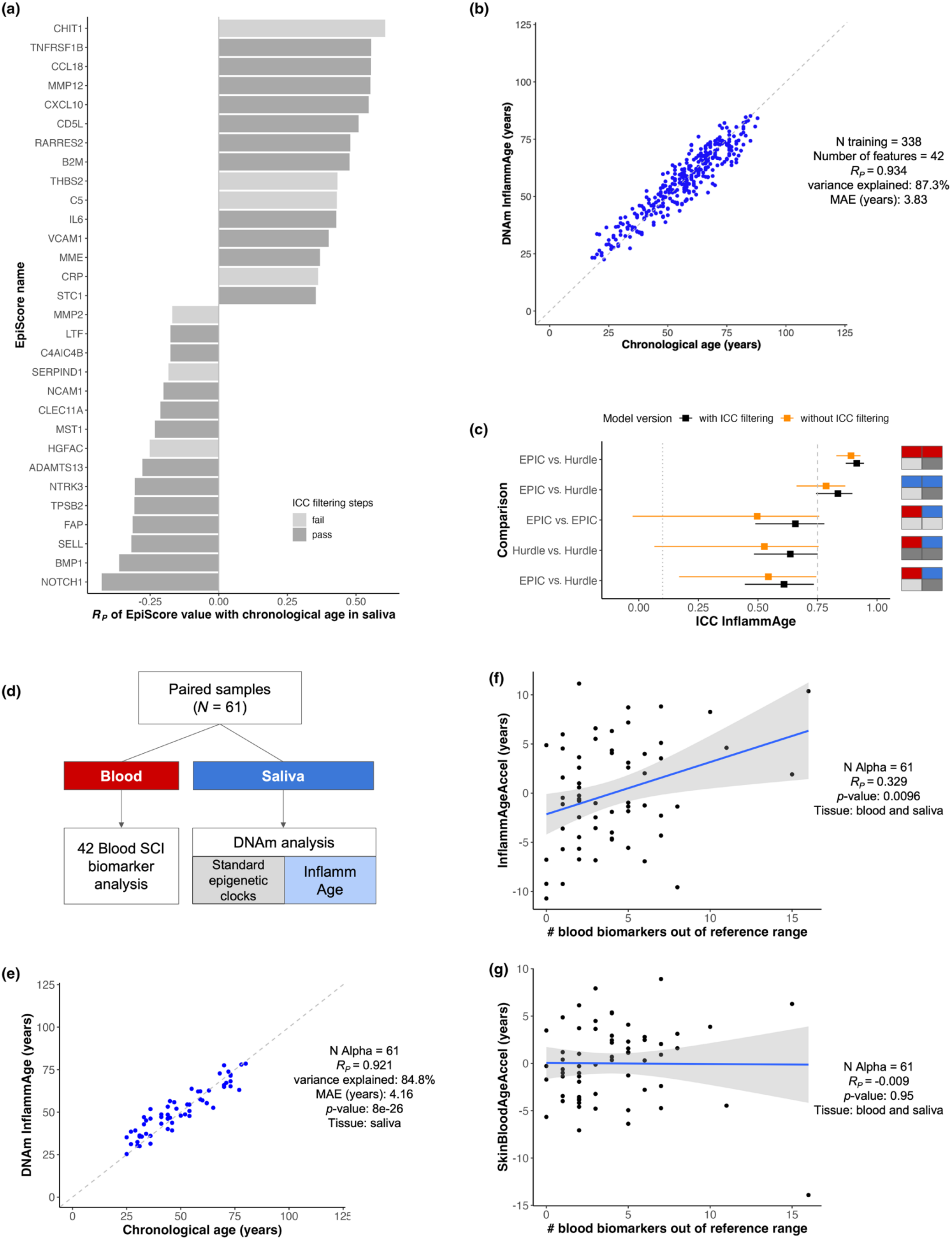
Train and test results of a saliva‐based DNAm biomarker for systemic chronic inflammation (InflammAge). (a) Barplot of top 15 positive and negative SCI DNAm EpiScore features significantly associated with chronological age (FDR‐adj. *p*‐value <0.05) in the saliva training dataset, see Figure S6 for the full list. Features passing all technical filtering steps (dark‐grey) were used to train InflammAge. *R*_*P*_, Pearson correlation coefficient. (b) Scatterplot of InflammAge performance (training dataset). (c) Reproducibility of InflammAge within and between tissues measured by intraclass correlation coefficient (ICC) in the reliability dataset (V2 samples), squares = mean, bars = 95% confidence interval. Values in black show improvement after filtering by ICC before training (framework steps 3 and 4). Orange = Results for a model without ICC‐based feature filtering. Red = blood, blue = saliva; light‐grey = EPIC, dark‐grey = Hurdle DNAm platform). (d–g) Testing InflammAge performance in the Alpha test dataset. (d) Cohort design (*N* = 61 individuals) with matched saliva DNAm and gold standard blood SCI biomarkers. (e) Scatterplot of InflammAge performance in the Alpha test dataset. (f, g) Scatterplots of association between SCI status in blood (quantified as number of SCI blood biomarkers outside of normal reference ranges in an individual) with (f) age‐adjusted InflammAge acceleration (InflammAgeAccel) and (g) age‐adjusted acceleration in the skin‐blood clock (SkinBloodAgeAccel) (Horvath et al., 2018). Blue lines = linear model fit, grey shading = confidence interval.

To train our InflammAge biomarker in saliva, we assembled a cohort with human saliva DNAm samples from published and in‐house data (training dataset, see Appendix S1). This dataset (*N* = 338, 27% female) covers the average adult human lifespan (age range of 18–88 years, median 58.5 years, mean = 56.8 years), including individuals from diverse genetic backgrounds (Table S3). In this cohort, we calculated the correlation (Pearson correlation coefficient, *R*_*P*_) of each EpiScore with chronological age in saliva (see Figure 2a for top 15 positively and negatively correlated and Figure S6 for all EpiScores). The biological assumption is that if the EpiScore for an inflammatory protein increases or decreases in the human population (i.e., cross‐sectionally) with age, then it is more likely to be a marker for SCI. We kept those features with associations with age in saliva that had *p*_*FDR*_ < 0.05 (step 5 of the framework, see Figure 1c).

This retained 63/125 EpiScores (dark grey in Figure S6); with 42 positively and 21 negatively correlated with age. EpiScores with positive correlation with age in saliva included well characterised pro‐inflammatory markers like TNFRSF1B (*R*_*P*_ = 0.56, *p*_*FDR*_ = 4.8 × 10^−27^), CCL18 (*R*_*P*_ = 0.56, *p*_*FDR*_ = 4.8 × 10^−27^), MMP12 (*R*_*P*_ = 0.55, *p*_*FDR*_ = 6.8 × 10^−27^), CXCL10 (*R*_*P*_ = 0.55, *p*_*FDR*_ = 2.8 × 10^−26^), or IL‐6 (*R*_*P*_ = 0.43, *p*_*FDR*_ = 1.7 × 10^−15^) (see Section 3). EpiScores with negative correlation with age in saliva included NOTCH1 (*R*_*P*_ = −0.43, *p*_*FDR*_ = 2.1 × 10^−15^), BMP1 (*R*_*P*_ = −0.36, *p*_*FDR*_ = 4.3 × 10^−11^), FAP (*R*_*P*_ = −0.31, *p*_*FDR*_ = 1.9 × 10^−8^) and NTRK3 (*R*_*P*_ = −0.31, *p*_*FDR*_ = 3.9 × 10^−8^). Positively age‐associated EpiScore genes were significantly enriched for inflammatory processes, cytokine activity, and response to stress in a gene ontology analysis (Figure S7a). Negatively age‐associated EpiScore genes were significantly associated with serine peptidase activity, humoral immune, and biotic stimulus response (Figure S7b). These results give confidence that the proteins quantified by the selected EpiScores are enriched for inflammatory and immune response pathways.

Next, we trained our DNA methylation biomarker (InflammAge) in our saliva training dataset using an elastic net algorithm (step 6 of the framework). We selected the 63 DNAm features enriched for inflammation‐related pathways as the input and chronological age as the outcome. In the training dataset, InflammAge predicted chronological age with a performance in range with gold standard epigenetic clocks (*R*_*P*_ = 0.93, median absolute error MAE = 3.83 years, Figure 2b). A total of 42 DNAm features (containing a total of 3788 unique CpG sites in the Hurdle DNAm platform) constitute the final InflammAge model. We calculated age‐adjusted InflammAge acceleration (InflammAgeAccel), which represents the difference between measured InflammAge and the average InflammAge expected for someone with the chronological age of the measured individual. A positive (>0) InflammAge acceleration should be associated with higher SCI when compared to other individuals of the population with the same chronological age (i.e., that person should be “inflammageing” faster). Saliva comprises a mix of immune cells, epithelial cells, and fibroblasts, and these proportions may change during ageing or disease (e.g., oral disease) (Aps et al., 2002). To study how the InflammAge biomarker may be affected by changes in saliva cell composition, we assessed the association of InflammAgeAccel with DNAm‐predicted cell type proportions (see Section 4). All these associations are non‐significant (Figure S8), which highlights that a higher InflammAgeAccel is not mainly driven by changes in cell composition. Of note, other gold standard blood‐ and buccal cell‐trained epigenetic clocks (skin‐blood clock (Horvath, 2013), multi‐tissue Horvath, Hannum, paediatric clock (PedBE, McEwen et al., 2019), PhenoAge, PCGrimAge (Higgins‐Chen et al., 2022), DunedinPACE) exhibited strong associations between age‐adjusted epigenetic age acceleration and the proportions of different cell types in saliva (Figure S9).

We then tested the performance of InflammAge in two independent datasets (step 7 of the framework). In the reliability dataset (V2 samples), reliability of InflammAge was good in saliva (ICC = 0.84 [0.74–0.90]) and excellent in blood (ICC = 0.91 [0.87–0.95]) (Figure 2c), showing that saliva DNAm biomarkers are reproducible when trained using our framework. Moderate reliability (ICC = 0.61 [0.44–0.73]; for saliva Hurdle vs. blood EPIC) between tissues was also observed, which highlights that even though InflammAge was trained in saliva DNAm samples, the results can be extrapolated to blood DNAm samples assessed on alternative platforms (Figures S4 and S5). Importantly, training an alternative InflammAge model using all the features associated with age as input but without filtering for ICC (i.e., skipping steps 3 and 4 in the framework) resulted in a less reliable biomarker with wide confidence intervals across all comparisons (Figure 2c). This validates the importance of the reliability dataset and steps 3 and 4 in our framework.

Finally, we tested the performance of InflammAge in the Alpha test dataset (see Appendix S1). This cohort was recruited for this work (*N* = 61, 46% female) and also broadly covers the average adult human lifespan (age range of 25–80 years, median = 46 years, mean = 49.6 years), including only self‐reported healthy individuals. Importantly, this dataset included matched saliva DNAm and blood SCI biomarkers (Figure 2d), which allows for assessing whether InflammAge is capturing changes in the SCI status of the individuals (besides tracking chronological age). InflammAge‐predicted chronological age with a correlation (*R*_*P*_) of 0.94 and MAE of 4.2 years (Figure 2e). Importantly, InflammAgeAccel was associated with the inflammatory biomarker CRP (*R*_*P*_ = 0.30, *p* = 0.02, Figure S10) and also associated with the total number of blood SCI biomarkers outside of reference ranges (*R*_*P*_ = 0.33, *p* = 0.01, Figure 2f). On the contrary, age‐adjusted epigenetic age acceleration of another saliva‐based epigenetic clock (skin‐blood clock, Horvath et al., 2018), was not correlated (*R*_*P*_ = −0.01, *p* = 0.95, Figure 2g). We further benchmarked performance against other gold standard epigenetic clocks including multi‐tissue Horvath, blood‐trained Hannum, PhenoAge, PCGrimAge, DunedinPACE, and the buccal‐swab trained PedBE clock (Figure S11 and Table S4). Only PhenoAgeAccel showed a significant and positive correlation with the number of blood SCI biomarkers (*R*_*P*_ = 0.26, *p* = 0.04) in the same direction as InflammAgeAccel, although with a smaller effect size (Figure S11f). This demonstrates the unique ability of InflammAge to capture SCI status from a saliva DNAm sample.

### 2.3 InflammAge is accelerated in SCI‐related clinical outcomes

Since saliva DNAm cohorts with mapped clinical outcomes are rare, we tested InflammAge in Generation Scotland (GS), one of the world's largest biobanks with access to blood DNAm samples linked to electronic health records (Table S5). GS has blood DNAm samples from 18,865 individuals (58.8% female) covering the adult human lifespan (age range of 17–99 years, median = 49.2 years, mean = 47.8 years).

InflammAge calculated in the GS dataset showed a strong correlation with chronological age, although an intercept shift was observed (i.e., a systematic underestimation of InflammAge in blood when compared to saliva DNAm samples; *R*_*P*_ = 0.904, MAE = 10.42 years; Figure S12). We decided to proceed to use GS as a test dataset using InflammAgeAccel, which as mentioned before is age‐adjusted and would correct for such a shift in the downstream analyses.

We tested the Spearman correlation coefficients (*R*_*S*_) between InflammAgeAccel and basic demographic and biochemistry variables (Table S6). Despite sex chromosome probes having been removed from all datasets in this study, InflammAgeAccel showed a significant difference when stratified by sex, where males had a mean InflammAgeAccel of 0.198 compared to −0.139 years in females (*t* test, *p* = 3.1 × 10^−4^). This is consistent with reports in the literature that highlight higher SCI baseline levels in males (Martínez de Toda et al., 2023). InflammAgeAccel (from samples taken between 2006 and 2011) was also associated with positive COVID‐19 disease diagnosis, ascertained from primary and secondary care records (hazard ratio HR = 1.24, 95% confidence interval [1.1–1.4], *p* = 3.1 × 10^−4^).

**FIGURE 3:**
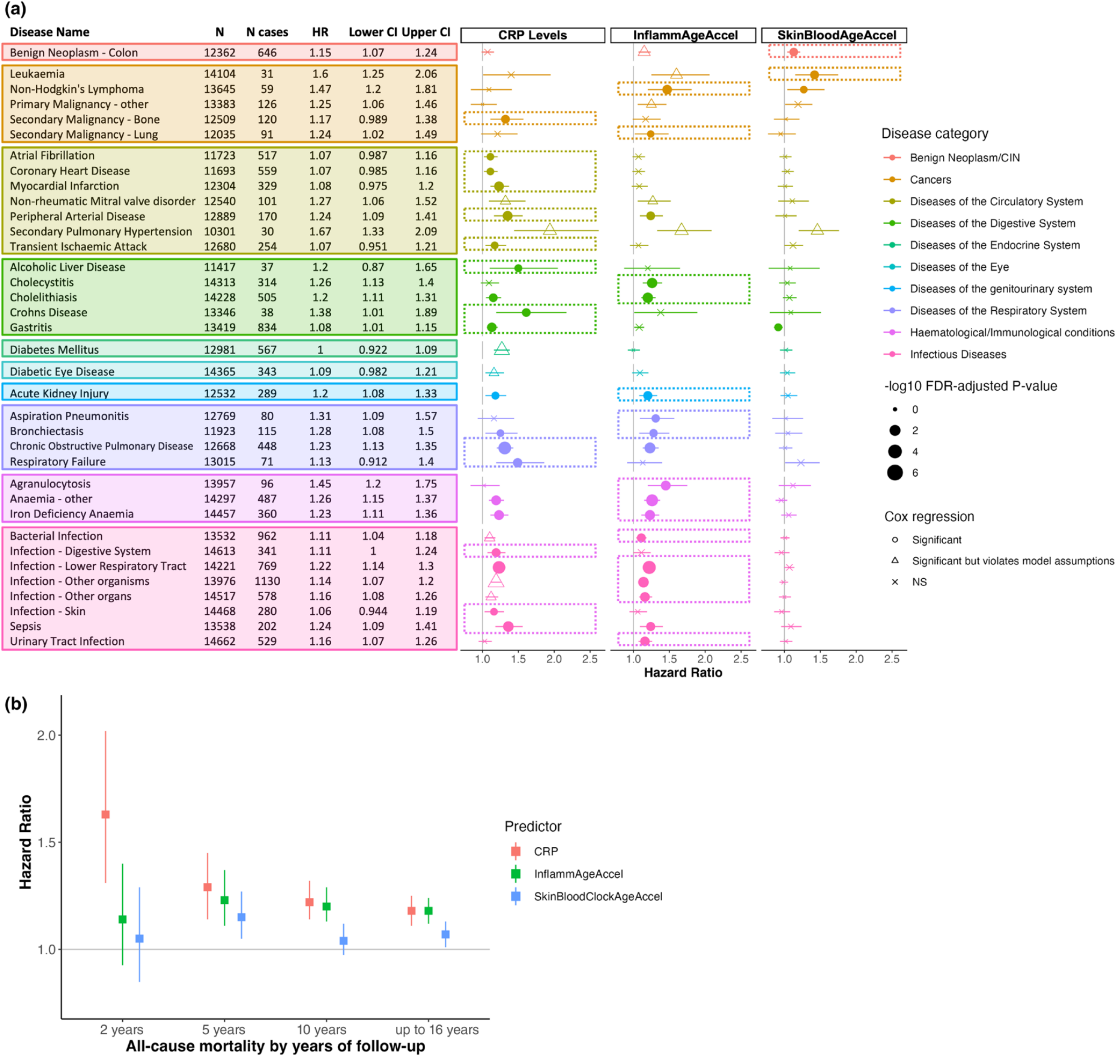
InflammAge acceleration performance in clinical endpoints compared to gold standard SCI markers in Generation Scotland. Incidence of clinical endpoint predicted for InflammAge acceleration (InflammAgeAccel), skin‐blood clock age acceleration (SkinBloodAgeAccel) and CRP using covariate‐adjusted Cox regression models. (a) Disease incidence Cox regression analysis. Displayed are 36 diseases with >30 cases in GS and association FDR‐adj. *p*‐value <0.05 for at least one predictor. The table shows hazard ratio (HR) and 95% confidence intervals (CI) per disease for InflammAgeAccel (full summary statistics in Table S6). The plot shows HR and 95% CI across all three predictors (best performed highlighted by dashed box). Colour = disease category (Kuan et al., 2023), shape = significance (NS, nonsignificant), filled = no model assumption violations. Point size is proportional to −log10 of FDR‐adj. *p*‐value. (b) Mortality prediction in GS for 2 (*N* = 66 events), 5 (*N* = 241 events), 10 years (*N* = 683 events) and the entire follow‐up period (*N* = 1031 events). Squares = HR, lines = 95% CI.

Next, we screened for an association between InflammAgeAccel and 308 diseases that were annotated using disease codes from Kuan et al. (2023). This was performed using Cox proportional hazards regression analyses that included demographic risk factors (age, sex, BMI, years of education, socioeconomic status, smoking pack‐years, and DNAm processing batch) as covariates. This allows us to assess if InflammAgeAccel adds significant value when modelling the risk of disease when compared with traditional risk factors. Figure 3a summarises results for 36 diseases with >30 cases that showed significant association across three biomarkers (InflammAgeAccel, SkinBloodAgeAccel and blood CRP—a gold standard inflammatory biomarker) with the best performing one highlighted per disease.

InflammAgeAccel was associated with the incidence of 18 diseases after multiple testing correction and without violation of the model assumptions (shown as filled circles in Figure 3a); 33 diseases were significant when using unadjusted *p*‐values (Table S7). The 18 hits included strong associations with the risk of developing diseases where SCI plays an important role in the aetiology, such as cancer (e.g., non‐Hodgkin's lymphoma, HR = 1.47 [1.20–1.81], *p*_*FDR*_ = 5.78 × 10^−4^), diseases of the circulatory system (e.g., peripheral arterial disease, HR = 1.24 [1.09–1.41], *p*_*FDR*_ = 3.37 × 10^−3^), diseases of the digestive system (e.g., cholecystitis, HR = 1.25 [1.13–1.40], *p*_*FDR*_ = 1.01 × 10^−4^), and diseases of the respiratory system (e.g., COPD, HR = 1.23 [1.13–1.35], *p*_*FDR*_ = 2.06 × 10^−5^). InflammAgeAccel outperformed CRP in 15 out of these 18 diseases (based on a comparison of FDR‐adj. *p*‐values). Furthermore, InflammAgeAccel outperformed the only widely available saliva‐based ageing DNA methylation biomarker (skin‐blood clock, SkinBloodAgeAccel) in all 18 diseases. CRP generally outperformed InflammAgeAccel in the “diseases of the circulatory system” category, and skin‐blood was mainly predictive for some cancer types. This confirms that InflammAge captures SCI‐related disease outcomes from an accessible tissue.

Covariate‐adjusted cox proportional hazards regression models were performed to assess the relationship between InflammAgeAccel and all‐cause mortality. This was assessed at intervals of 2 years (*N* = 66 events), 5 years (*N* = 241 events), 10 years (*N* = 683 events), and the entire follow‐up period (up to 16 years, *N* = 1031 events). Except for the 2‐year interval, there was a significant relationship between InflammAgeAccel and all‐cause mortality (Figure 3b). The performance of InflammAgeAccel was comparable to that of blood‐based CRP measurements and outperformed the skin‐blood clock when predicting mortality within 5 years (HR_CRP_ = 1.29 [1.14–1.45], HR_InflammAgeAccel_ = 1.23 [1.11–1.37], HR_SkinBloodAgeAccel_ = 1.15 [1.05–1.27]), within 10 years (HR_CRP_ = 1.22 [1.14–1.32], HR_InflammAgeAccel_ = 1.20 [1.13–1.29], HR_SkinBloodAgeAccel_ = 1.04 [0.97–1.12]), and the entire follow‐up period (up to 16 years: HR_CRP_ = 1.18 [1.11–1.25], HR_InflammAgeAccel_ = 1.18 [1.12–1.24], HR_SkinBloodAgeAccel_ = 1.07 [1.01–1.13]). When compared to blood‐based epigenetic clocks, InflammAgeAccel performed in the range of PhenoAgeAccel, while GrimAgeAccel and DunedinPACE outperformed all other predictors (Figure S13). This is expected since InflammAge was not trained specifically to predict all‐cause mortality, but rather to capture the SCI component of human ageing.

### 2.4 InflammAge acceleration is associated with lifestyle factors

Lifestyle factors can have a profound influence on human healthspan, lifespan, and SCI status (Furman et al., 2019). We tested the association of InflammAgeAccel with lifestyle factors such as self‐reported smoking status, alcohol consumption, physical activity, and dietary variables in the GS cohort.

**FIGURE 4:**
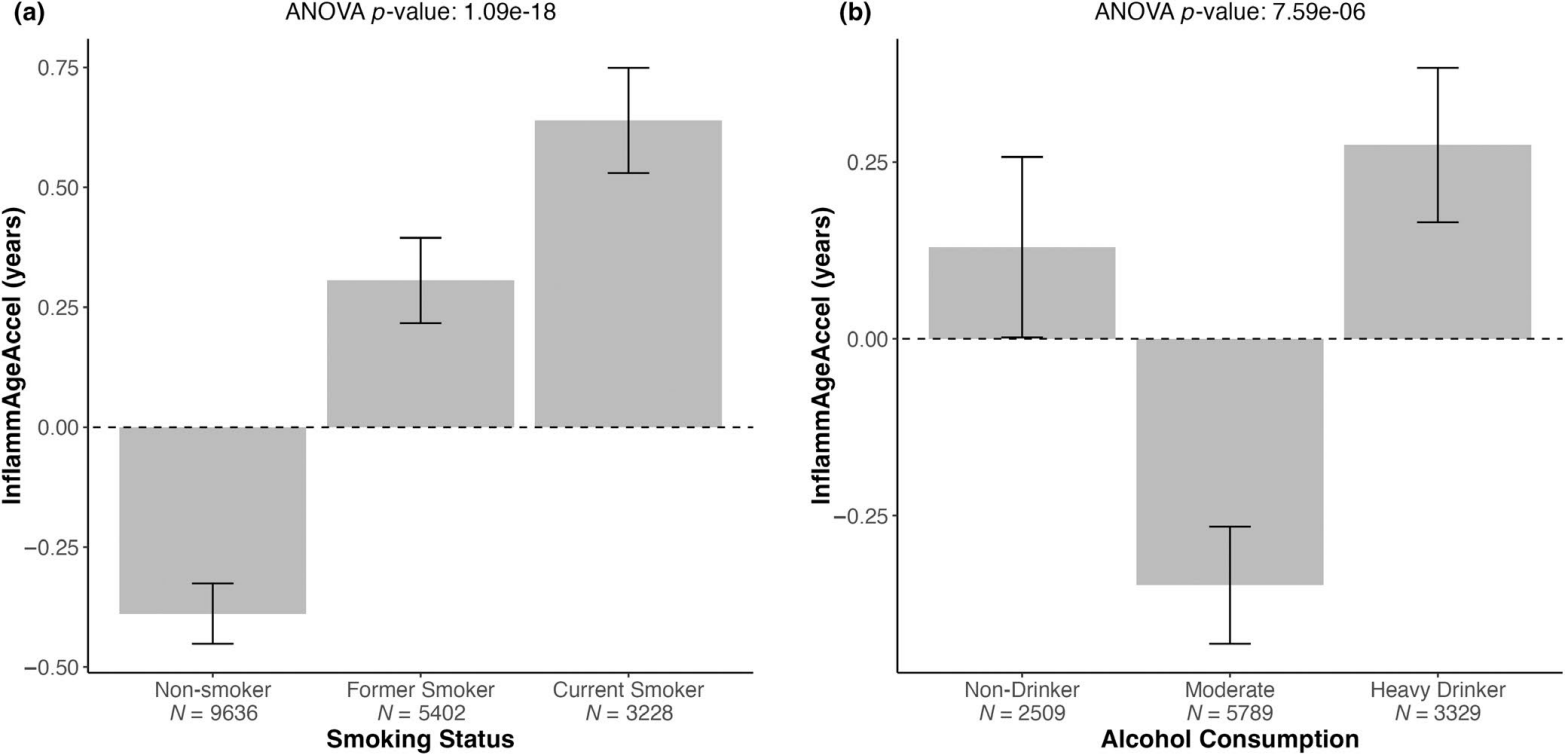
InflammAge acceleration (InflammAgeAccel) associations with smoking status and alcohol intake in GS. Barplot with ANOVA *p*‐value for (a) self‐reported smoking status, and (b) self‐reported alcohol consumption in the week before the blood sample for DNAm measurement was taken. Plots show mean InflammAgeAccel per group, error bars, standard errors of the mean.

Smoking status was significantly associated with InflammAgeAccel (ANOVA *p* = 1.1 × 10^−18^) in a dose–response manner, with non‐smokers showing the lowest acceleration (ex‐smoker vs. non‐smoker: Diff = −0.70 [−0.95 to 0.44], Tukey adj. *p*‐value *p*_*T*_ = 3.2 × 10^−8^; smoker vs. non‐smoker: Diff = 1.03 [0.73–1.33], *p*_*T*_ = 3.2 × 10^−8^) (Figure 4a). For alcohol consumption, InflammAgeAccel was higher in heavy (≥14 units/week) compared to moderate drinkers (<14 units/week; Diff = 0.62 [0.30–0.95], *p*_*T*_ = 1.8 × 10^−5^), while lower in moderate compared to non‐drinkers (Diff = −0.48 [−0.83 to 0.12], *p*_*T*_ = 4.4 × 10^−3^) (Figure 4b).

Self‐reported physical activity was not associated with InflammAge acceleration (*R*_*S*_ = −0.01, *p* = 0.16). This was unexpected, given the links between exercise and reductions in systemic chronic inflammation (Furman et al., 2019). However, this could be due to the nature of self‐reported exercise data, which tends to be less accurate than wearable‐based data.

When analysing the association of dietary consumption patterns with InflammAgeAccel, we ran linear models adjusted for the same confounding factors used in the disease outcome analysis (Table 1). An intake of oily fish at any frequency was significantly associated with lower InflammAgeAccel compared to never having oily fish (Effect size = −0.31, standard error = 0.13, *p* = 0.02). Intake of other dietary constituents at any frequency compared to never showed no significant difference when adjusted for confounding factors. However, individual dose groups showed significance in the unadjusted ANOVA model (see Table S8). This included lower InflammAgeAccel for individuals with a higher intake of brown bread (5–6 days/week vs. never: Diff = −0.94 [−1.66 to 0.23], *p*_*T*_ = 2 × 10^−3^), higher fruit (daily vs. once per week: Diff = −0.84 [−1.51 to 0.17], *p*_*T*_ = 4.4 × 10^−3^) and higher green vegetables intake (5–6 days/week vs. never: Diff = −1.08 [−2.00 to 0.17], *p*_*T*_ = 8.8 × 10^−3^). On the contrary, a higher intake of meat showed a trend towards higher InflammAgeAccel (intake daily vs. < once per month: Diff = 1.15 [0.17–2.12], *p*_*T*_ = 9.7 × 10^−3^) although this was not observed across all dietary consumption amounts. These effects are consistent with reports for other blood‐based epigenetic clocks (Levine et al., 2018; Lu et al., 2019; Quach et al., 2017).

**TABLE 1:** Association of InflammAgeAccel with dietary variables in GS.

Dietary category	Effect size	Standard error	P‐value
Oily fish	−0.305	0.134	0.023
Dairy	−1.017	0.664	0.126
Brown bread	−0.315	0.216	0.145
Other fish	0.232	0.213	0.275
Eggs	−0.223	0.358	0.534
Liver	−0.053	0.105	0.613
Green vegetables	−0.147	0.302	0.627
Poultry	0.120	0.269	0.656
Other meat	0.089	0.251	0.724
Fruit	−0.150	0.470	0.749
Other vegetables	−0.128	0.570	0.823

*Note*: Summary statistics for covariate‐adjusted linear models testing association between InflammAgeAccel and dietary variables (ever versus never categories, collected at baseline during GS participant recruitment). Full summary statistics of all ANOVA comparisons in Table S5.

Overall, our results suggest that InflammAge has potential to monitor the impact of lifestyle factors on SCI.

### 2.5 InflammAge recapitulates immunosenescent cell type composition in blood

**FIGURE 5:**
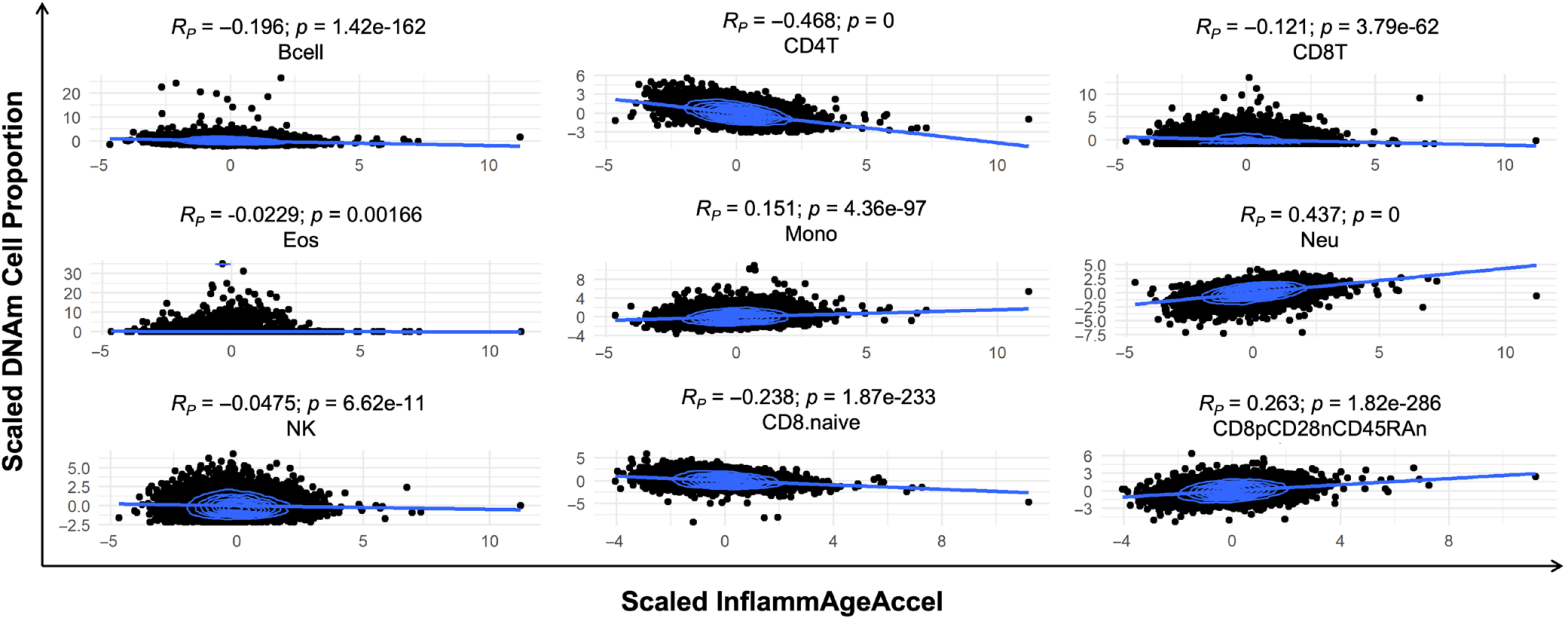
InflammAge acceleration (InflammAgeAccel) recapitulates immunosenescent cell type composition. Scatterplot of InflammAgeAccel and blood cell type proportions estimated from GS blood DNAm data (scaled and centred around zero with standard deviation = 1). Bcell, B‐cell; CD4T, CD4+ T‐cells; CD8.naive, naive CD8+ T‐cells; CD8pCD28nCD45RAn, memory/effector CD8+ T‐cells; CD8T, CD8+ T‐cells; Eos, eosinophils; Mono, monocytes; Neu, neutrophils; NK, natural killer cells; *p*, *p*‐value; *R*_*P*_, pearson correlation coefficient.

We performed cell type deconvolution in blood DNAm data from GS and calculated the correlation of InflammAgeAccel with the estimated proportions of each cell type. InflammAgeAccel was positively correlated with monocytes, neutrophils and CD8 + CD28‐CD45RA‐ T cells (memory/effector CD8+ T cells, Tomiyama et al., 2002). In contrast, InflammAgeAccel was negatively correlated with B cells, CD4+ T cells, total CD8+ T cells, and naïve CD8+ T cells. Eosinophils and NK cells showed almost no change with InflammAgeAccel (Figure 5). This is consistent with changes observed in the immune system during human ageing and immunosenescence (Saule et al., 2006). Thus, individuals that have positive InflammAgeAccel (InflammAge results higher than expected) display a more immunosenescent profile.

## 3 DISCUSSION

Preventative healthcare and interventions that target ageing directly can have a profound health and economic impact on our societies. A slowdown in ageing that results in an increase in human life expectancy by 1 year is understood to be worth US$38 trillion (Scott et al., 2021). This can be transformational for healthcare systems and patients who currently live carrying the burden of multi‐morbidity. In order to understand the ageing process and the risk of developing age‐related diseases, accessible and actionable biomarkers need to be developed and made widely available.

In this work, we created a framework that facilitates the creation of novel saliva‐based DNA methylation biomarkers. Saliva as an accessible and non‐invasive sample type comprises ~80% immune cells and ~20% epithelial buccal cells and fibroblasts. This overlap in cell composition substantiates the strong correlations (*r* ~ 0.97) between saliva and blood DNAm patterns (Nishitani et al., 2023), and alongside the results of this study, supports the case for saliva DNAm as a clinically meaningful and reproducible data type to build biomarkers. In fact, the first ever‐developed epigenetic clock, which explained 73% variance in chronological age using two CpG sites, was developed using saliva DNA methylation data (Bocklandt et al., 2011). Recent studies have also leveraged the saliva's non‐invasiveness to develop the first DNA methylation age predictor specialised for paediatric samples (McEwen et al., 2019), or sought to adapt epigenetic clocks trained in blood for application in saliva by accounting for differences in cell‐type composition (Galkin et al., 2021). Given that most biological data in humans, including DNAm data, has been generated in blood samples, the ability to transfer biological information from blood to saliva DNAm opens the door for preventative healthcare and population screening applications at scale, including at‐home testing and reaching isolated populations.

We illustrated the value of our biomarker discovery framework by creating InflammAge, a novel saliva‐based DNAm biomarker for SCI. InflammAge was built bottom‐up by selecting DNAm features that are associated with chronic inflammatory markers, which allows for biological interpretability of the results. This may also benefit clinical trials to improve patient stratification since individuals may age differently, that is, have different “ageotypes” that personalised interventions should recognise. Using DNAm EpiScores that aggregate multiple CpG probes, we also benefit from dimensionality reduction as a de‐noising mechanism, which has been reported to significantly improve the reliability of epigenetic clocks (Higgins‐Chen et al., 2022).

During feature selection for InflammAge, we uncovered DNAm predictors of blood inflammatory proteins that change consistently during ageing. Importantly, many of these EpiScores were trained on protein levels adjusted for age, sex, cohort‐specific variables, and potential genetic protein quantitative trait loci (pQTL) effects (Gadd et al., 2022); which requires careful interpretation when comparing them to direct protein concentration measurements and drawing biological conclusions from these features. Nevertheless, EpiScores that show age‐associated increase include several examples of pro‐inflammatory proteins, such as IL‐6, a classic biomarker for inflammageing, TNFRSF1B, one of the receptors of TNF‐alpha, or CXCL10, a cytokine induced by TNF‐α and IFN‐γ.

Monocytes and macrophages are considered key effector cellular populations for inflammageing. Traditionally, M1 macrophages are thought to be pro‐inflammatory while M2 macrophages are anti‐inflammatory and involved in tissue repair. Interestingly, M1/M2 macrophage populations change during human ageing (Costantini et al., 2018) and we observed evidence that InflammAge may be identifying this phenomenon, for example, through an increase in the DNAm proxy for proteins like CCL18, which causes M2 macrophage maturation, or MMP12, a matrix metalloprotease secreted by M2 macrophages. Relatedly, NOTCH1, whose inhibition increases M2 macrophage polarisation (Wang et al., 2010), was the top EpiScore negatively associated with age. Notch signalling has been proposed as a target in chronic inflammatory diseases, and further studies investigating this phenomenon may benefit from the application of InflammAge and/or related EpiScores to enhance translation of findings.

Thanks to the rich electronic health record data available in GS we performed the most comprehensive screening to assess the ability of a new epigenetic clock to predict clinical outcomes. We demonstrated that InflammAgeAccel captures associations with all‐cause mortality, disease outcomes, and lifestyle factors; in many cases outperforming CRP, a widely‐used gold standard SCI biomarker. For most of the cancer outcomes, InflammAgeAccel showed a higher hazard ratio (e.g., for non‐Hodgkin's lymphoma (NHL)) when compared to CRP. A meta‐analysis of 17 studies identified that elevated levels of several inflammatory biomarkers (such as TNF‐α) are associated with a higher risk of developing NHL, potentially implicating them in the aetiology of the disease (Makgoeng et al., 2018). Albeit violation of some model assumptions, InflammAgeAccel improved results from CRP also for leukaemia. A recent single‐cell multi‐omics analysis identified a role for chronic inflammation as a driver of leukemic evolution (Rodríguez‐Meira et al., 2023). InflammAgeAccel similarly outperformed CRP in the disease categories “haematological/immunological conditions” and “infectious diseases”. The latter could be reflective of the ability of InflammAge to quantify immunosenescence in blood and the link between an increase in SCI and the decreased ability of the immune system to fight infection with age. While CRP was better at capturing all‐cause mortality in the short term (2 years), InflammAgeAccel‐matched CRP in the longer term (up to 16 years), perhaps indicating that InflammAge captures the medium to long‐term (as opposed to acute) risk of disease.

This study has several limitations. First, the reliability (*N* = 83) and saliva DNAm training (*N* = 338) datasets were relatively small. As more saliva DNAm data from consenting individuals becomes available, our biomarker discovery framework and performance of new versions of InflammAge are likely to improve. Interestingly, unlike what we observed in blood, InflammAgeAccel was not significantly associated with cell composition in saliva in our analysis; which could be related to the fact that InflammAge was trained using blood DNAm proxies. These conclusions should be further tested in larger and independent datasets. Additionally, it would be important to study whether oral disease can confound the results for InflammAge in future studies. Furthermore, the current saliva DNAm datasets do not have linked mortality or disease outcome data, which precluded us from a saliva‐only training and testing set‐up, necessitating training on chronological age and testing in a large blood DNAm dataset like GS instead. A direct comparison against blood‐trained epigenetic clocks (like PhenoAge, DunedinPACE, or GrimAge) should be performed against a blood‐based InflammAge biomarker to avoid bias based on the training tissue type. Additionally, sex differences were observed for our biomarker, with the male population displaying a higher InflammAge acceleration. Given that organs and systems display sex‐specific ageing processes (including immune system ageing), investigating this in more detail (or even training sex‐specific biomarkers) is necessary. When looking at the proposed extension of the NIH‐FDA Biomarkers EndpointS and other Tools (BEST) classification framework recently suggested by the “Biomarkers of Ageing Consortium” (Moqri et al., 2023), InflammAge is best suited to be classified as a predictive or prognostic biomarker. Understanding how InflammAge changes longitudinally and how responsive it is upon intervention is currently underway. For example, InflammAgeAccel showed an improvement for individuals with high SCI at baseline in a 3‐month nutraceutical intervention study (McGee et al., 2024).

Future work will continue to explore if our saliva‐based DNAm framework can be used to deconvolute the ageing process and quantify other hallmarks of ageing with accessible, biologically interpretable biomarkers. Ultimately, we envision a saliva‐based and cost‐effective DNAm test that is able to personalise follow‐up diagnostics and interventions and that becomes the entry point for a preventative healthcare journey for the global asymptomatic population.

## 4 MATERIALS AND METHODS

### 4.1 Hurdle DNAm platform

We compiled CpG probes associated with relevant health and disease phenotypes from the EWAS catalogue (Battram et al., 2022) and EWAS Atlas (Li et al., 2019) (accessed March 2022). After harmonisation and filtering for genome‐wide significance (*p* < 3.6 × 10^−8^), a core set of high‐confidence associations was formed by intersecting studies and including up to 1000 CpG probes per study (Figure S1a). Additionally, relevant literature reporting EWAS results for phenotypes of interest was manually curated, resulting in the inclusion of approximately 30,000 CpG probes from 212 publications (Figure S1b). These probes were deployed in a cost‐effective Illumina methylation array termed the Hurdle DNAm platform.

### 4.2 Genomic annotation of CpG sites

We annotated the genomic location of the Hurdle CpG sites using the manifest from the Illumina EPIC 850 k array (mapped to hg19, Infinium MethylationEPIC v1.0 B5 Manifest file). We obtained gene distribution categories using the UCSC_RefGene_Group annotation column: Promoter <− c(“TSS200”, “TSS1500”), Gene_body <‐ c(“Body”, “UTR”, “Exon”), Intergenic (no annotation present); where a probe overlapped multiple genes in both the Promoter and Gene_body category, it was annotated as a Promoter in this analysis. We defined CpG Island distribution categories using the Relation_to_UCSC_CpG_Island annotation column: CpG_Islands <‐ c(“Island”), CpG_Shores <‐ c(“N_Shore”, “S_Shore”), “>2 kb from Islands” (no or “Shelf” annotation). We defined DNase Hypersensitivity categories based on having a named DHS in the DNase_Hypersensitivity_NAME annotation column. We then plotted the percentage of CpG probes with an annotation in each category compared to all Hurdle or all EPIC probes.

### 4.3 Dataset description

Construction and processing of the training, reliability, and test datasets is described in Appendix S1.

### 4.4 DNA methylation data processing pipeline

This applies to all DNAm data presented in this manuscript except the GS cohort. Data processing was performed using RStudio (Version: 2023.09.1+494). IDAT files were processed using a custom pipeline leveraging the *minfi* (Aryee et al., 2014) and *SeSaMe* (Zhou et al., 2018) packages. The pipeline uses *noob* background correction and BMIQ within‐array normalisation as previously described (Martin‐Herranz et al., 2019). We removed cross‐reactive, SNP‐ and sex chromosome‐overlapping probes. Samples with overall call rates <70% or low mean intensity values were removed from analyses. We subset probes to a set of ~30 K included in the Hurdle DNAm platform before or after normalisation as indicated. GS DNAm data was processed as described in Appendix S1.

### 4.5 Reliability analyses

ICCs were calculated using a customised version of the *dupicc* function of the *ENmix* R package version 1.36.08 (Xu & Taylor, 2021) for a single‐rater, absolute‐agreement, two‐way random‐effects model (type = agreement, model = c(twoway), unit = single). Only probes present in both platforms (EPIC & Hurdle) after quality control were used for probe level ICC (29,472). V1 samples of the reliability dataset were used for ICC calculations at feature selection (steps 3 + 4 in the framework), and V2 samples for testing of the final InflammAge biomarker (step 7) to avoid data leakage. EpiScores and InflammAge calculated in each dataset were used as input for the *dupicc* function for feature and biomarker level ICC.

### 4.6 Calculating EpiScores in saliva DNAm data

EpiScores were selected by literature review and calculated using coefficients and methods originally provided (Aslibekyan et al., 2018; Gadd et al., 2022; Hillary et al., 2020; Ligthart et al., 2016; Stevenson et al., 2020, 2021). For 109 EpiScores containing inflammatory proteins measured in the Olink and SOMAscan platforms (Gadd et al., 2022), the function *scale* (center = TRUE, scale = TRUE) from the *base* R package was used to normalise the beta‐values across samples in the dataset before calculating the EpiScores.

### 4.7 EpiScore gene enrichment analysis

Gene Ontology (GO)‐term enrichment was performed using the g:Profiler platform (Kolberg et al., 2023). Gene names of EpiScore proteins selected after ICC filtering were analysed in two sets (positively/negatively associated with chronological age in saliva). All annotated genes were used as background for enrichment. Input gene names were ranked by using the absolute Pearson correlation coefficient with age in saliva.

### 4.8 Training the InflammAge biomarker

Elastic net regression was used to train a model that would predict chronological age (y_fit, dependent/output variable) using the selected SCI‐related DNAm features (x_fit, independent variables). We used 10‐fold cross‐validation and alpha = 0.5 as implemented in the *cv.glmnet* function in the *glmnet* R package (Friedman et al., 2010):

$$\text{cvfit}\_\text{model}<-\mathrm{cv}.\text{glmnet}\left(x\_\mathrm{fit},y\_\mathrm{fit},\text{alpha}=0.5\right).$$

We calculated age‐adjusted InflammAge acceleration (InflammAgeAccel) as the residuals from this linear regression model:

DNAm_InflammAge~Chronological_age.

### 4.9 Saliva cell type composition analysis

Cell type composition of the saliva cohorts was estimated from DNA methylation data with the EpiDISH (Epigenetic Dissection of Intra‐Sample Heterogeneity) R package, using the Robust Partial Correlations‐RPC method (Teschendorff et al., 2017).

### 4.10 Benchmarking against other epigenetic clocks

This applies to all epigenetic clocks calculated in this manuscript except for the GS cohort samples. We ran a suite of gold standard epigenetic clocks in our saliva training and testing cohorts to benchmark the performance of InflammAge. The epigenetic clocks were calculated using the dnaMethyAge R package (Wang, 2021; Wang et al., 2024). These included: SkinBlood: skin‐blood clock (Horvath et al., 2018); Horvath: multi‐tissue Horvath clock (Horvath, 2013); Hannum: Hannum clock (Hannum et al., 2013); PedBE: paediatric clock (McEwen et al., 2019); PhenoAge: PhenoAge clock (Levine et al., 2018); PCGrimAge: principal components GrimAge clock (Higgins‐Chen et al., 2022); DunedinPACE: DunedinPACE clock (Belsky et al., 2022).

For analysis in the GS dataset, DNAm data was uploaded to the UCLA clock calculator (https://dnamage.genetics.ucla.edu/home), described in Horvath (2013) to calculate GrimAge and PhenoAge acceleration. Dunedin PACE was calculated using the PACEprojector function in the DunedinPACE R package (https://github.com/danbelsky/DunedinPACE).

### 4.11 Disease association analysis in GS

A series of Cox proportional hazards regression models were run to relate InflammAgeAccel (residual of InflammAge ~ chronological age) to the risk of developing 308 diseases, as coded by Kuan et al. (2023). Additional covariates included age at blood draw, sex, log‐transformed BMI, years of education, socioeconomic status (Scottish Index of Multiple Deprivation), smoking pack years, and DNAm processing batch. Where the proportion of females with an incident diagnosis for each disease was >90% (or <10%), we stratified the model to analyse females (males) only. Given some diseases more likely occur at certain periods of life (e.g., dementia after the age of 65 years), we calculated age of diagnosis for each disease, extracted age of cases at the 2.5 and 97.5 percentiles and filtered age at the event or censoring to fall within this window to retain a set of controls with similar profiles to the cases. Censoring was marked by being unaffected by the disease and either surviving to the latest point of linkage (April 2022) or time to death during follow‐up. Regression assumptions were examined by tests of the Schoenfeld residuals, with p‐value <0.05 indicating a model violation. These analyses were repeated with log‐transformed measured CRP and other age‐adjusted epigenetic age acceleration (SkinBloodAgeAccel, PhenoAgeAccel, GrimAgeAccel, DunedinPACE) metrics for diseases with >30 cases (to preserve participant anonymity and reduce the risk of models not converging).

In addition to the diseases listed in Kuan et al. (2023), we examined the relationship between InflammAgeAccel and a COVID‐19 disease diagnosis (see Appendix S1).

### 4.12 Lifestyle factor association analysis in GS

Alcohol consumption was binned into non‐drinker (0 units in the last week), moderate (<14 units) and heavy drinker groups (> = 14 units) and filtered to individuals reporting intake as their usual weekly intake. ANOVA tests were performed on smoking and alcohol groups, followed by Post‐hoc Tukey tests to assess two‐group relationships. Physical activity reported as metabolic equivalents (MET) minutes was scaled and Spearman correlation with scaled InflammAge acceleration was calculated.

Dietary intake data included self‐reported information on frequency of brown bread, fruit, green vegetables, other vegetables, liver, poultry, other meat (non‐poultry), eggs, dairy, oily fish, and other fish (non‐oily). Two types of analyses were performed for dietary intake data:

1. An “ever/never” diet variable was derived per multi‐level diet variable. Effect sizes and *p*‐values were obtained from linear models adjusted with covariates (Table 1):

$$\begin{array}{c} \text{scale}\left(\text{InflammAgeAccel}\right)\sim \mathrm{age}+\mathrm{sex}+\log\left(\mathrm{BMI}\right)+\text{education}\_\text{years}\\ \;+\text{scale}\left(\text{deprivation}\_\mathrm{rank}\right)+\text{smoking}\_\text{status}\\ \;+\text{DNAm}\_\text{processing}\_\text{batch}+\text{diet}\_\text{variable} \end{array}$$

2 To test associations between diet levels (doses), ANOVA and post‐hoc Tukey tests were performed as described (Table S7).

### 4.13 Cell type composition analysis in GS

White blood cell proportions were estimated using meffil's implementation of the Houseman algorithm (Houseman et al., 2012) with Reinius et al.'s peripheral blood reference data (Reinius et al., 2012). Additional measures (CD8pCD28nCD45RAn) were obtained from the Horvath clock output (https://dnamage.genetics.ucla.edu/home, Horvath, 2013). Blood cell proportions were scaled to mean 0 and standard deviation 1 using the *scale* function. Correlation between scaled InflammAgeAccel and scaled cell proportions was calculated for each cell type using Pearson correlation coefficient. Figures were plotted using *ggplot2* in R.

## AUTHOR CONTRIBUTIONS

LJS, TPC, DLM, HJ, EZ, and DEMH carried out analyses. LJS, TPC, TMS, and DEMH devised the study and secured funding. KMG, JS, AC, AMM, TJ, and JML performed study design and recruitment for the reliability dataset and contributed data. VJ, DK, NT, ES, MG, RT, and the Hurdle bio‐infrastructure team performed and supervised tech work and logistics. WJH, VO, KW, CEB, and REM advised on the experimental and InflammAge validation strategy. WJH assisted in the generation of manuscript figures. LJS, TPC, HJ, TMS, and DEMH wrote the first draft and all authors revised and approved the manuscript.

## CONFLICT OF INTEREST STATEMENT

LJS, HJ, VJ, DK, NT, EZ, ES, MG, and the Hurdle bio‐infrastructure team are employees and TPC, RT, TMS, and DEMH are co‐founders and shareholders at Chronomics Ltd., a company focused on multi‐omics and epigenetic biomarker development and diagnostic infrastructure. VO and KW are employees at Bayer Consumer Care AG. CEB is an employee at Bayer HealthCare LLC. JML and WJH declare their roles as consultants for Bayer Consumer Care AG. Both report receiving consultancy fees from Bayer Consumer Care AG during the preparation of the study. Bayer Consumer Care AG financially supported this work. REM acts as a scientific consultant for Optima Partners and is an advisor to the Epigenetic Clock Development Foundation. All other authors declare no competing interests.

## Supporting information


Appendix S1.

## Data Availability

Public training datasets (listed in Table S3) are available in GEO (Edgar et al., 2002) and ArrayExpress (Parkinson et al., 2007). Data from Hurdle's users or study participants cannot be made publicly available due to consent limitations, but requests for research collaboration can be submitted to the corresponding authors. Generation Scotland allows researchers to apply for access to their data through their collaboration proposal process. Further details can be found at https://www.ed.ac.uk/generation‐scotland/for‐researchers/access. We invite academics to contact us directly about running InflammAge in their cohorts.

## Appendix

The Hurdle bio‐infrastructure team includes: Pierric Descamps, Emily Taylor, Chuck Paiusi, Leona Mc Girr, Ollie Philpott, Ian Robinson, Hector Watson, Ana Magalia, Dave Russell, and David Woods, all employees at Chronomics Ltd., London, UK.
