# Supplementary material for: A novel framework to build saliva‐based DNA methylation biomarkers: Quantifying systemic chronic inflammation as a case study
Source: Aging Cell. 2025 Jan 30;24(4):e14444. doi: 10.1111/acel.14444 (PMC11984670; doi:10.1111/acel.14444)
Supplement: Supplementary file 1 — Appendix S1. [file ACEL-24-e14444-s001.zip › 3_Schmunk_et_al_2024_SupplementaryInformation.pdf]

Supplementary Information to

A novel framework to build saliva-based DNA methylation biomarkers: quantifying systemic chronic inflammation as a case study

Lisa J. Schmunk[1*], Toby P. Call[1], Daniel L. McCartney[2], Hira Javaid[1], Waylon J. Hastings[3], Vanja Jovicevic[1], Dragoljub Kojadinović[1], Natacha Tomkinson[1], Eliska Zlamalova[1], Kirsty C. McGee[4], Jack Sullivan[4], Archie Campbell[2], Andrew M McIntosh[2,5], Veronika Óvári[6], Karl Wishart[6], Christian E. Behrens[7], Emma Stone[1], Miloš Gavrilov[1], Rob Thompson[1], Hurdle bio-infrastructure team[1], Thomas Jackson[4], Janet M. Lord[4,8], Thomas M. Stubbs[1], Riccardo E. Marioni[2], Daniel E. Martin-Herranz[1*]

[1]Hurdle.bio / Chronomics Ltd., London, UK.
[2]Centre for Genomic and Experimental Medicine, Institute of Genetics and Cancer, University of Edinburgh, Edinburgh, UK.
[3]Department of Psychiatry and Behavioral Sciences, Tulane University School of Medicine, New Orleans, LA, USA.
[4]MRC-Versus Arthritis Centre for Musculoskeletal Ageing Research, Institute of Inflammation and Ageing, University of Birmingham, Birmingham, UK.
[5]Division of Psychiatry, Centre for Clinical Brain Sciences, University of Edinburgh, Edinburgh, UK.
[6]Bayer Consumer Care AG, Basel, Switzerland.
[7]Bayer HealthCare LLC, Whippany NJ, USA.
[8]NIHR Birmingham Biomedical Research Centre, University Hospitals Birmingham, Birmingham, UK.

*Corresponding authors: lisa.schmunk@hurdle.bio, daniel@hurdle.bio

This document includes:

- **Supplementary Methods:**
  - Note 1

- **Supplementary Figures**
  - Figures S1-S14

- **Supplementary Tables**
  - Tables S1-S3, S5-S6
  - Legends for Supplementary Tables S4, S7 and S8. Table S4, S7 and S8 are supplied in the accompanying .xlsx spreadsheet.

Supplementary Methods - Note 1

Training dataset

We created a training dataset with saliva genome-wide DNA methylation (DNAm) samples (N=338, **Table S3**) by combining published datasets and in-house saliva DNAm samples from UK-based individuals who provided informed consent for their pseudoanonymised data being used for research purposes. Only data from individuals aged 18 and above was used. Where cohorts indicated disease status, only samples from healthy controls were used.

Reliability dataset

The reliability dataset was generated as part of an independent study with healthy older adults aged ≥60 years (McGee et al., 2024). The study was approved by the UK Health Research Authority and the London-Bromley Research Ethics Committee (Reference: 22/PR/0698) and was conducted in accordance with the Declaration of Helsinki, with informed and written consent obtained from all participants prior to recruitment into the study. Saliva and venous blood samples of 83 adults were collected at two time points (baseline = visit 1 = V1 and week 12 = visit 2 = V2) with age 72.24 ± 6.46 years; BMI: 26.62 ± 3.99 kg/m^2; 31 males, 52 females (for full design of the DNAm data generated see **Figure S14**). Peripheral venous blood samples were acquired by venipuncture into vacutainers (BD Biosciences, Oxford, UK) containing EDTA and stored at -80°C until DNA extraction. Saliva samples were collected for DNA methylation analysis using Hurdle's sample collection kit (ORAgene DNA Saliva Collection kit; OG-600; DNA Genotek) and stored at 4°C prior to analysis. Genomic DNA from both saliva and whole blood were extracted and processed at Eurofins Genomics, Denmark. Bisulfite conversion was performed, samples split into two and processed in parallel on the EPIC v1.0 array and the Hurdle DNAm platform, creating technical replicates used for ICC calculation.

Test dataset: Alpha cohort

A matched blood SCI biomarkers and saliva DNAm dataset was collected as part of a Hurdle human study of UK-based individuals who provided written informed consent for their pseudoanonymised data being used for research purposes. A cohort of 61 participants between ages 18-80 remained after filtering for exclusion criteria and quality control for DNAm and blood results. Exclusion criteria were: acute infection in the past 6 weeks (including COVID-19 or influenza), prescription of immunosuppressant drugs, diagnosed chronic diseases affecting inflammatory status, heavy smokers (>20/day), harmful drinking habits (>35 units/week for women, >50 units/week for men), heavy exercise or significant diet changes prior to sampling. Saliva and venous blood samples were collected in the morning of the same day. Saliva samples were self-collected using Hurdle's sample collection kit. Peripheral venous blood samples were acquired by venipuncture into vacutainers containing EDTA, lithium heparin (LH) or z-serum clotting activator. Serum was isolated from vacutainers after incubation at room temperature for 30 minutes and centrifugation at 1620 x g for 10 minutes at room temperature and stored at -80°C. Plasma was isolated from LH or EDTA vacutainers by centrifugation at 461 x g for 8 minutes at room temperature and stored at -80°C. DNA methylation was measured from saliva samples using the EPIC v1.0 array at Eurofins Genomics, Denmark. Cytokine levels were quantified at Affinity Labs, London, UK. Albumin, complement C4, hsCRP (Siemens Advia 1800), and cortisol (Siemens Advia Centaur XP) were quantified in serum. IL-6, IL-1β, TNF-α, IFN-γ, IL-10, IL-8/CCL-8, IL-17, SAA (Mesoscale Discovery V Plex assay), CXCL-9, CXCL-1/GROα, GDF-15, TNFSF10/TRAIL, PAPP-A, CCL-11 (Biotechne ELISA), HbA1c, Full Blood Count: WBC, NEUT, MONO, LYM, ESO,

BASO, RBC, HB, HCT, MCV, MCH, MCHC, RDW, PLT, MPV (Siemens Advia 560) were quantified in plasma.

Test dataset: Generation Scotland (GS) cohort
Generation Scotland is a family-based cohort consisting of individuals aged 17 to 99 years, living across Scotland. Participants were recruited from individuals registered at GP surgeries who were then asked to invite first degree relatives to join the study, resulting in 23,960 individuals for whom genetic, clinical, lifestyle, and sociodemographic information are available.

DNA methylation was profiled in four sets between 2016 and 2022 using the EPIC v1.0 array. For all sets, IDAT files were read into R using functions within *minfi* v.1.20.2 – 1.42.0 (Aryee et al., 2014). Quality control (QC) was applied to each set separately. Briefly, outliers were removed based on visual inspection of a plot of the log median intensity of the methylated versus unmethylated signal for each array (set 1). *ShinyMethyl*'s control probe plots were then inspected to identify outliers. Next, samples for which the sex predicted from the methylation data (based on the difference between the median copy number intensity for the Y chromosome and the median copy number intensity for the X chromosome) did not match the recorded sex were removed, with multi-dimensional scaling (MDS) plots generated and inspected for any additional sample outliers. The *pfilter* function within *wateRmelon* v.1.18.0 (Pidsley et al., 2013) was used to remove: (i) samples where ≥ 1% of CpGs had a detection *p*-value > 0.05; (ii) probes with a beadcount of < 3 in > 5% samples; and (iii) probes for which ≥ 0.5% of samples had a detection *p*-value > 0.05. QC of the DNA methylation data produced in sets 2-4 was carried out using both *Meffil* vs.1.1.0 and 1.1.2 (Min, Hemani, Smith, Relton, & Suderman, 2018) and *shinyMethyl* versions 1.14.0 and 1.30.0 (Fortin, Fertig, & Hansen, 2014). *Meffil* was used to perform dye-bias and background correction using the "noob" method. Samples were excluded if they were 1) affected by a strong dye bias or issues affecting bisulphite conversion (using default thresholds); 2) had median methylated signal intensity greater than three standard deviations lower than expected; or 3) had a different methylation-predicted sex to the self-reported sex. Deviations between methylation-predicted sex and self-reported sex were also assessed using *shinyMethyl*'s sex prediction function, which uses a different methodology to *Meffil*. *ShinyMethyl* was additionally used to plot the output of all control probes to permit the detection of outliers by visual inspection. Following these sample removal steps, *Meffil* was used to filter poor-performing samples and probes. Samples were removed if they: had > 0.5% CpG sites with a detection *p*-value > 0.01. Once the poor-performing samples had been removed, *Meffil* was re-run on the remaining dataset to identify poor-performing probes. These were defined as probes with a beadcount of < 3 in > 5% samples and/or > 1% samples with a detection *p*-value > 0.01. X and Y chromosome probes were removed. Quality-controlled DNA methylation data from the four datasets were combined and jointly normalised using the *dasen* method (Pidsley et al., 2013) (N_{Set1}=5,055, N_{Set2}=458, N_{Set3}=4,374, N_{Set4}=8,978).

DNAm InflammAge was calculated and mapped to phenotypic and clinical outcome information for 18,865 volunteers in GS. Clinical outcomes were obtained from linkage to primary care (GP) data and secondary care (General Acute Inpatient and Day Case) electronic health records from the Scottish Morbidity Record 01 (SMR01).

COVID-19 association analysis in GS
We examined the relationship between InflammAge acceleration and a COVID-19 disease diagnosis, ascertained from primary and secondary care records. Cases were defined based on primary care Read codes 1JX1., 65PW1, A7951, 8CAO., 8CAO1, 9N312, and 4J3R1 (derived

from Scottish Clinical Information Management in Practice coding (*System COVID-19 Codes – Available March 2020 Coding – Primary Care Informatics*, 2020), and secondary ICD10 codes U071 and U072. Logistic regression, adjusting for the same covariates as above, was used to analyse this relationship. Individuals who died without a prior COVID-19 diagnosis were removed prior to the analysis.

Supplementary References

1. Aryee, M. J., Jaffe, A. E., Corrada-Bravo, H., Ladd-Acosta, C., Feinberg, A. P., Hansen, K. D., & Irizarry, R. A. (2014). Minfi: A flexible and comprehensive Bioconductor package for the analysis of Infinium DNA methylation microarrays. *Bioinformatics*, *30*(10), 1363–1369. https://doi.org/10.1093/bioinformatics/btu049
2. Fortin, J.-P., Fertig, E., & Hansen, K. (2014). shinyMethyl: Interactive quality control of Illumina 450k DNA methylation arrays in R. *F1000Research, 3,* 175. https://doi.org/10.12688/f1000research.4680.2
3. McGee, K. C., Sullivan, J., Hazeldine, J., Schmunk, L. J., Martin-Herranz, D. E., Jackson, T., & Lord, J. M. (2024). A combination nutritional supplement reduces DNA methylation age only in older adults with a raised epigenetic age. GeroScience. https://doi.org/10.1007/s11357-024-01138-8
4. Min, J. L., Hemani, G., Smith, G. D., Relton, C. L., & Suderman, M. (2018). Meffil: efficient normalization and analysis of very large DNA methylation datasets. *Bioinformatics, 34*(23), 3983–3989. https://doi.org/10.1093/bioinformatics/bty476
5. Pidsley, R., Wong, C. C. Y., Volta, M., Lunnon, K., Mill, J., & Schalkwyk, L. C. (2013). A data-driven approach to preprocessing Illumina 450K methylation array data. *BMC Genomics, 14*(1), 293. https://doi.org/10.1186/1471-2164-14-293

Supplementary Figures

Supplementary Figure 1

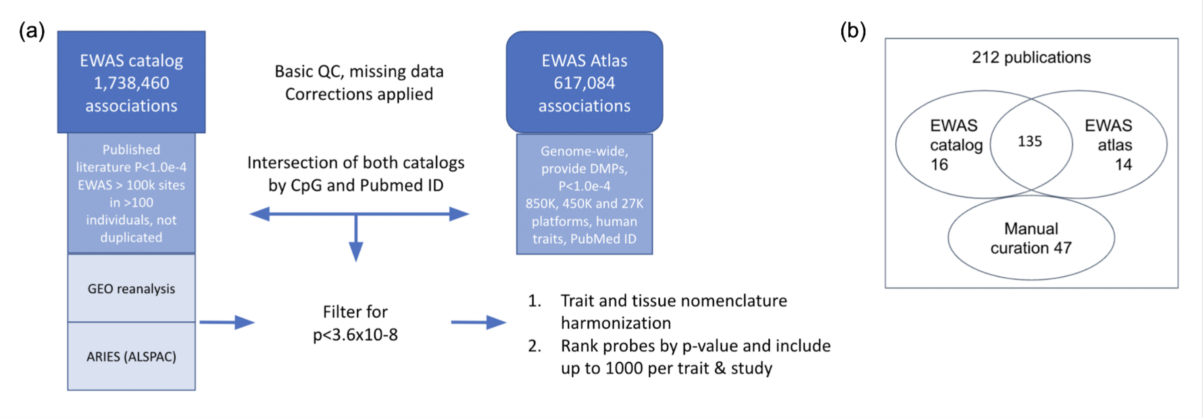

Figure S1 Hurdle DNAm platform design. (a) Diagram of CpG site selection strategy and (b) summary of publication sources included in the Hurdle DNAm platform.

Supplementary Figure 2

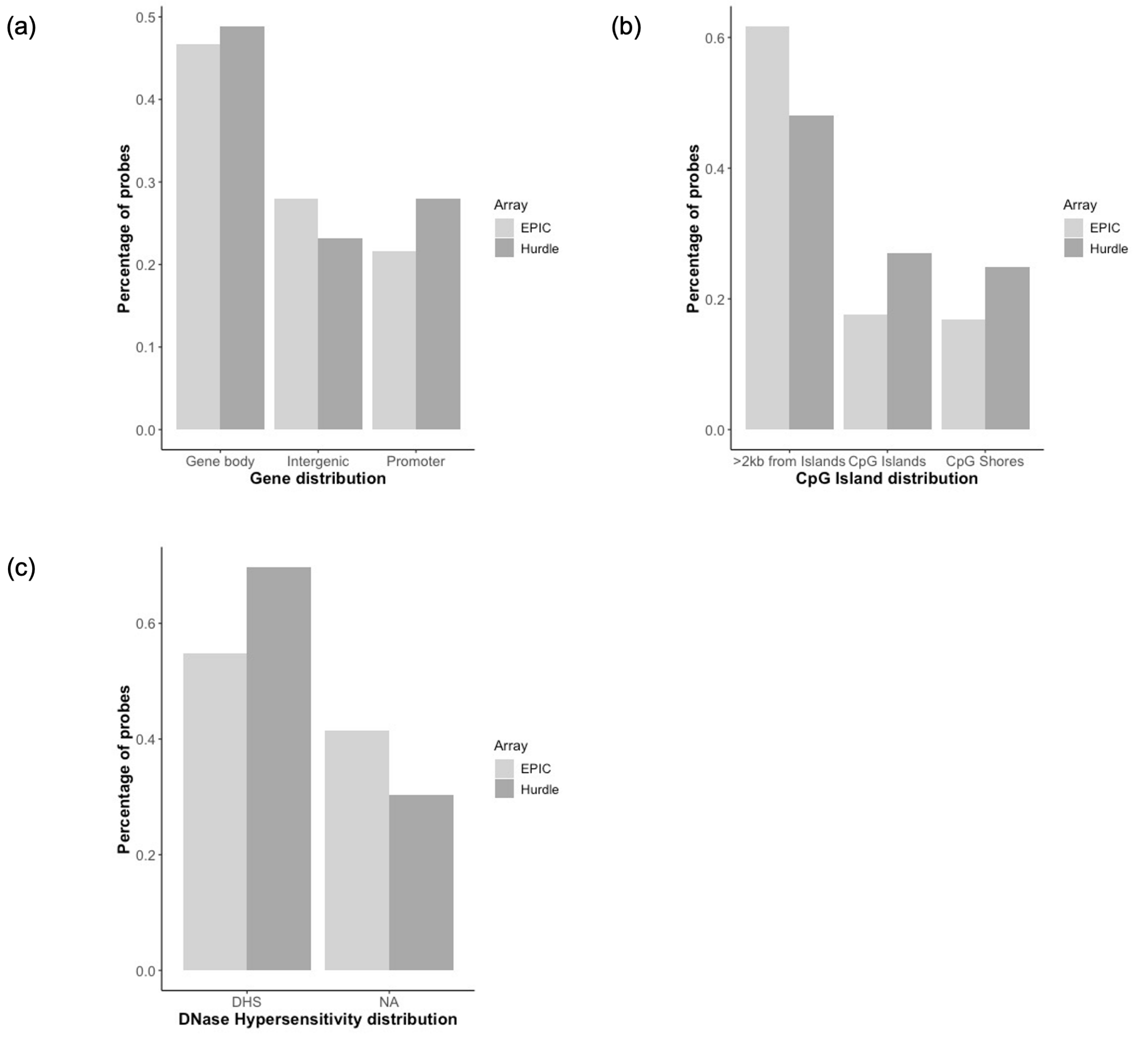

Figure S2 Genomic location of CpG sites (probes) included in the EPIC (light grey) and the Hurdle (dark grey) DNAm platforms. DHS: DNase Hypersensitivity Sites.

Supplementary Figure 3

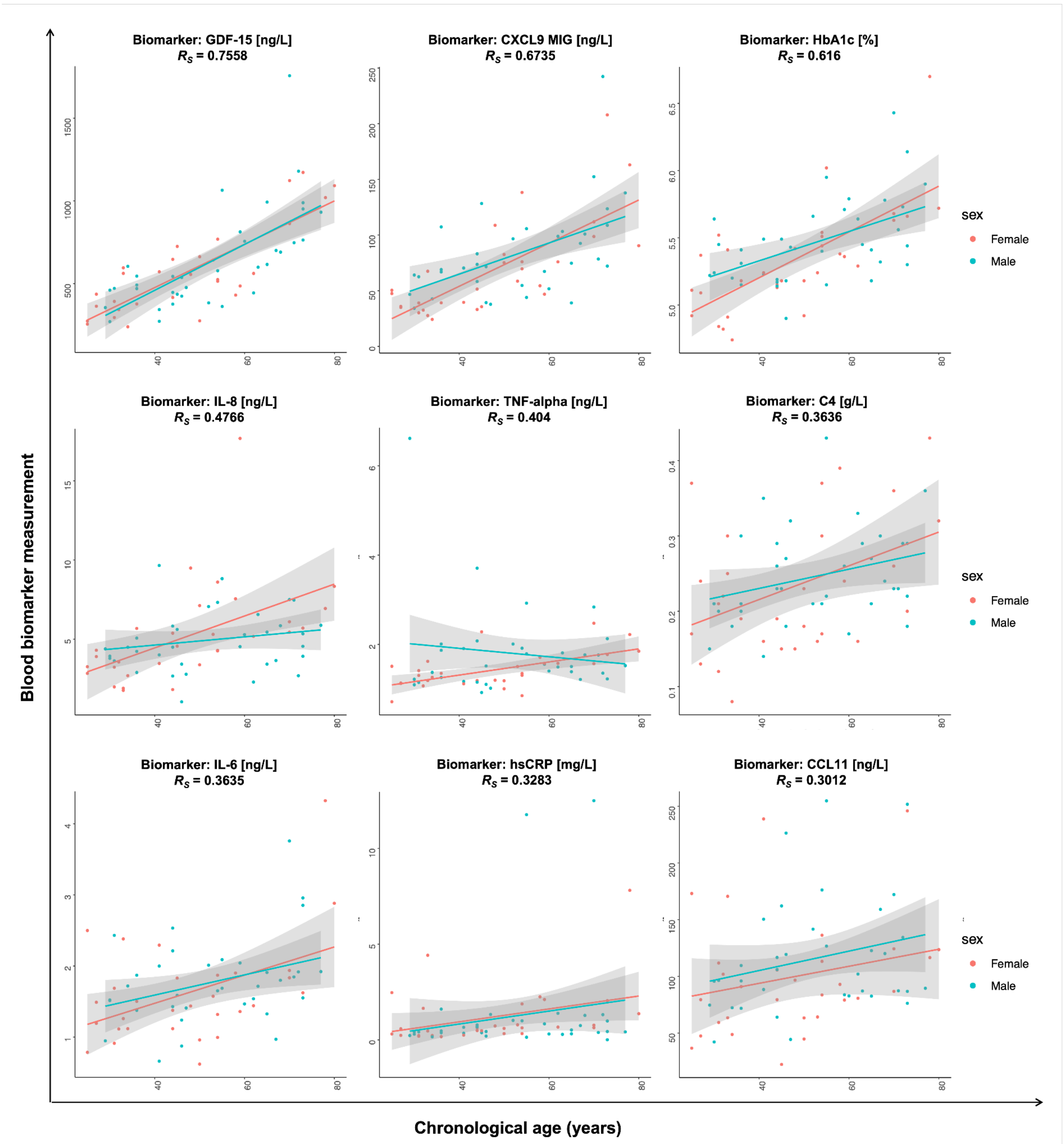

Figure S3 Blood biomarker associations with chronological age in the Alpha test dataset. Scatter plots with Spearman correlation coefficients (R_S) for the association of 9 blood biomarkers measured from blood serum or plasma in the Alpha test dataset (see **Supp. Methods - Note 1**). From top left to bottom right: GDF-15, CXCL9, HbA1c%, IL-8, TNF-α, C4, IL-6, CRP, CCL11.

Supplementary Figure 4

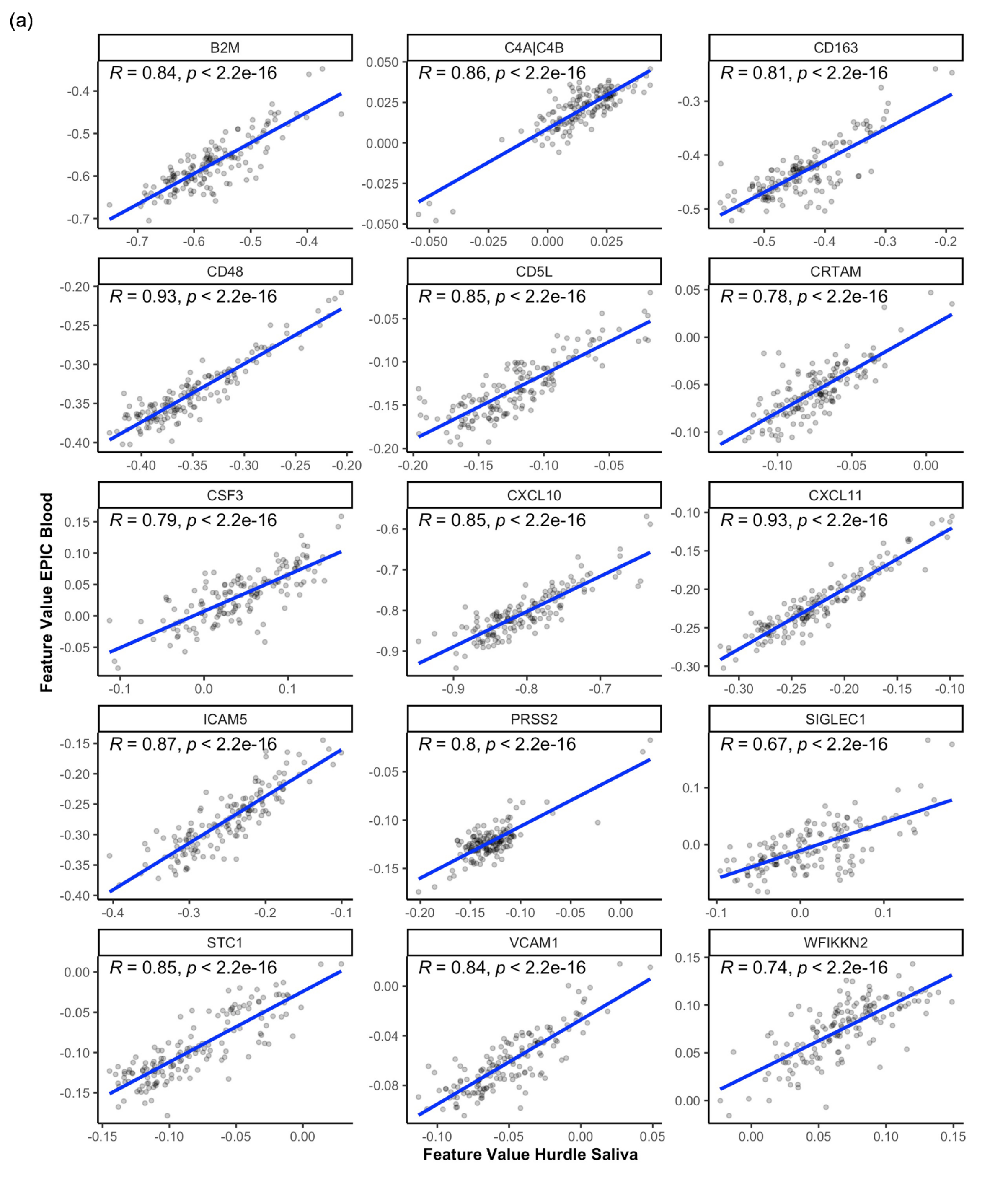

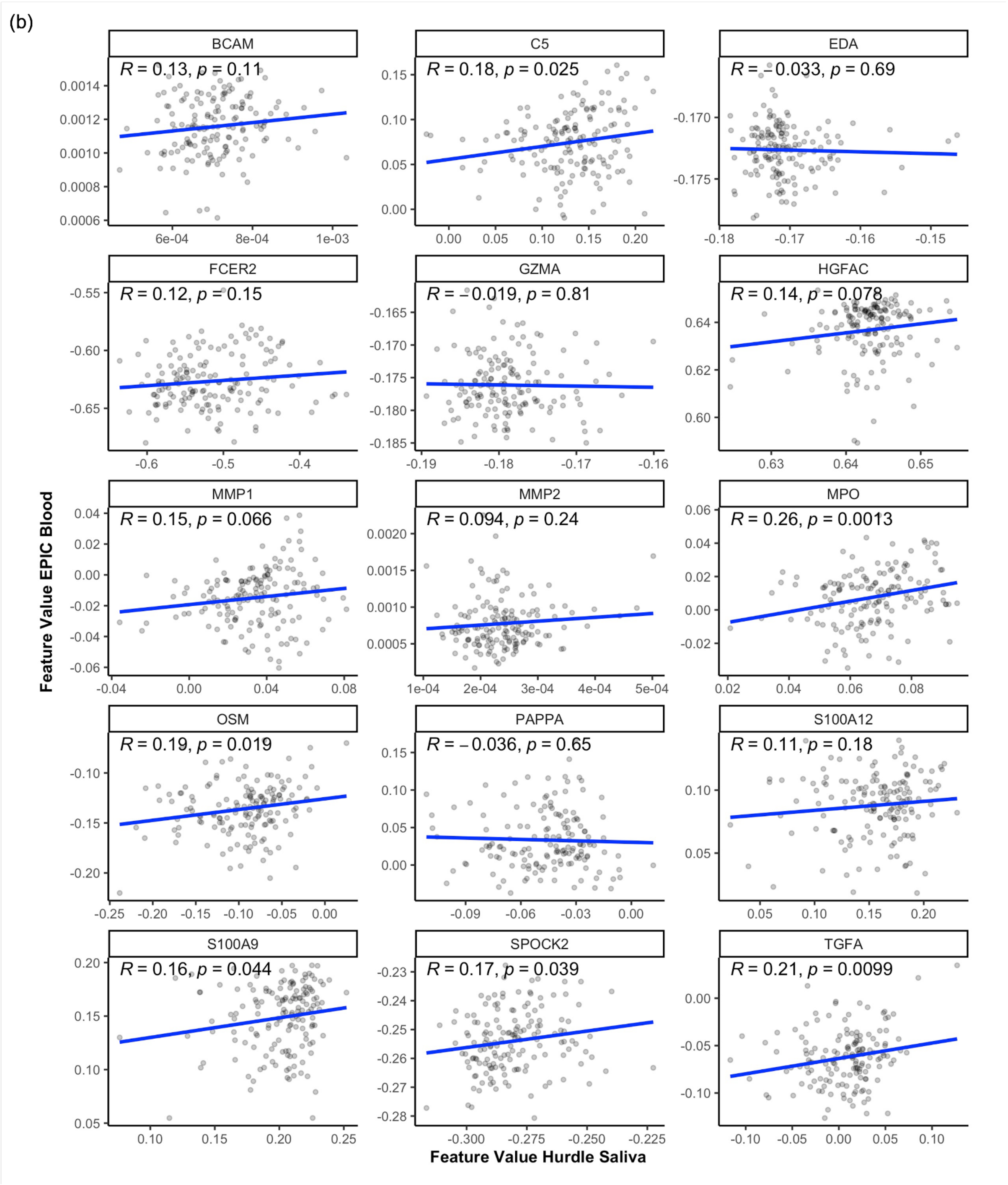

Figure S4 Cross-tissue correlations for EpiScore features in the reliability dataset. DNAm EpiScore values were calculated between matched blood EPIC and saliva Hurdle samples. (a) Scatter plots with Pearson correlation coefficients *R* for 15 EpiScores with the highest ICC values between blood EPIC and saliva Hurdle. (b) Scatter plots with *R* for 15 EpiScores with the lowest ICC values. *p:* unadjusted p-value.

Supplementary Figure 5

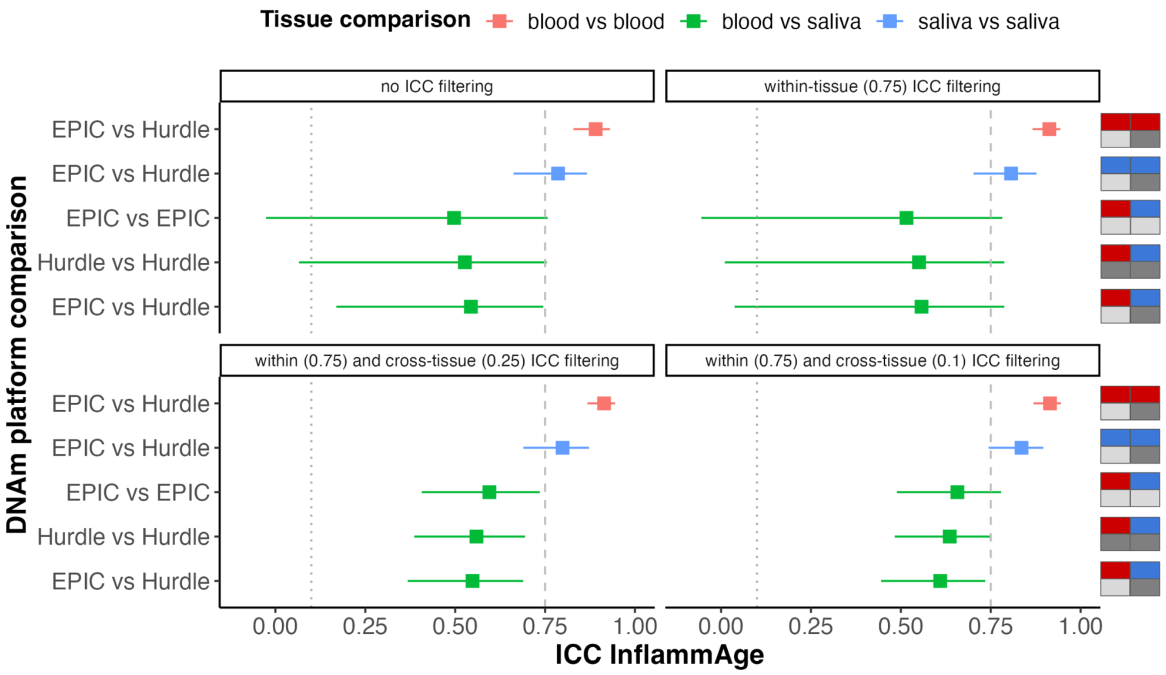

Figure S5 InflammAge ICC comparisons across model versions. InflammAge was trained with either no EpiScore ICC filtering applied before training (top left); EpiScores filtered within tissues for icc_EbHb>0.75, icc_EsHs>0.75 (equivalent to step 4, top right); EpiScores filtered within tissues for icc_EbHb>0.75, icc_EsHs>0.75 and across tissues for icc_EbEs>0.25, icc_HbHs>0.25 and icc_EbHs>0.25 (bottom left); or EpiScores filtered within tissues for icc_EbHb>0.75, icc_EsHs>0.75, icc_EbEs>0.10, icc_HbHs>0.10, icc_EbHs>0.10 (steps 3+4, bottom right). E= EPIC, H= Hurdle DNAm platform, b = blood, s = saliva. The dotted and dash lines at 0.1 and 0.75, respectively, indicate ICC filtering criteria chosen for the final InflammAge model. ICC InflammAge after training was calculated using the reliability dataset (V2 samples).

Supplementary Figure 6

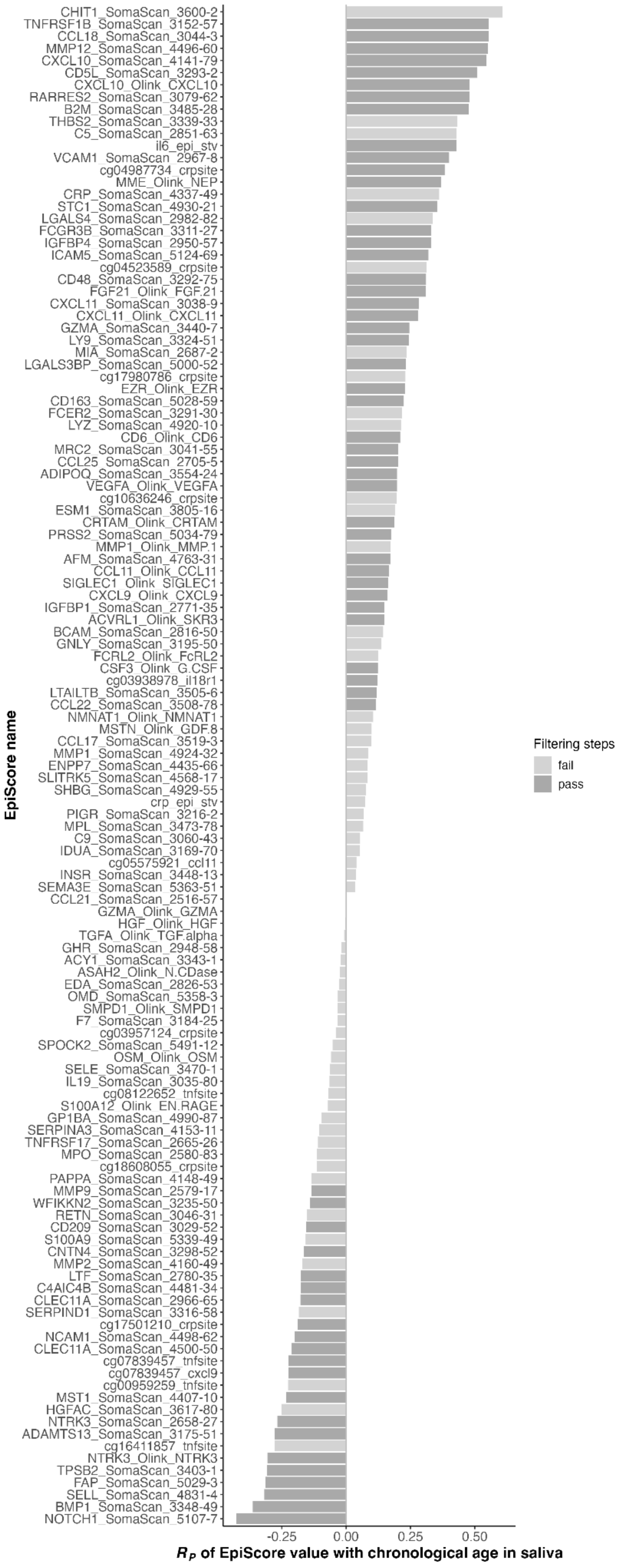

Figure S6 Barplot of Pearson correlation of 125 DNAm EpiScore features with chronological age in the saliva training dataset. Features passing all technical filtering steps (dark-grey) were used to train InflammAge. *R*$_P$=Pearson correlation coefficient.

Supplementary Figure 7

(a)

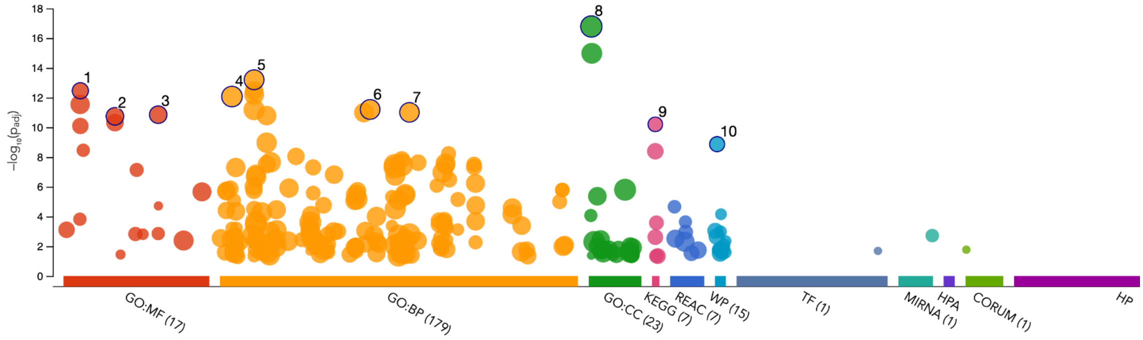

ID	Source	Term ID	Term Name	p_{adj} (query_...
1	GO:MF	GO:0005125	cytokine activity	3.475×10^{-13}
2	GO:MF	GO:0030546	signaling receptor activator activity	1.822×10^{-11}
3	GO:MF	GO:0048018	receptor ligand activity	1.412×10^{-11}
4	GO:BP	GO:0002376	immune system process	8.514×10^{-13}
5	GO:BP	GO:0006952	defense response	6.319×10^{-14}
6	GO:BP	GO:0044419	biological process involved in interspecies interaction between organisms	6.283×10^{-12}
7	GO:BP	GO:0051707	response to other organism	9.855×10^{-12}
8	GO:CC	GO:0005576	extracellular region	1.655×10^{-17}
9	KEGG	KEGG:04061	Viral protein interaction with cytokine and cytokine receptor	6.315×10^{-11}
10	WP	WP:WP5095	Overview of proinflammatory and profibrotic mediators	1.363×10^{-9}

(b)

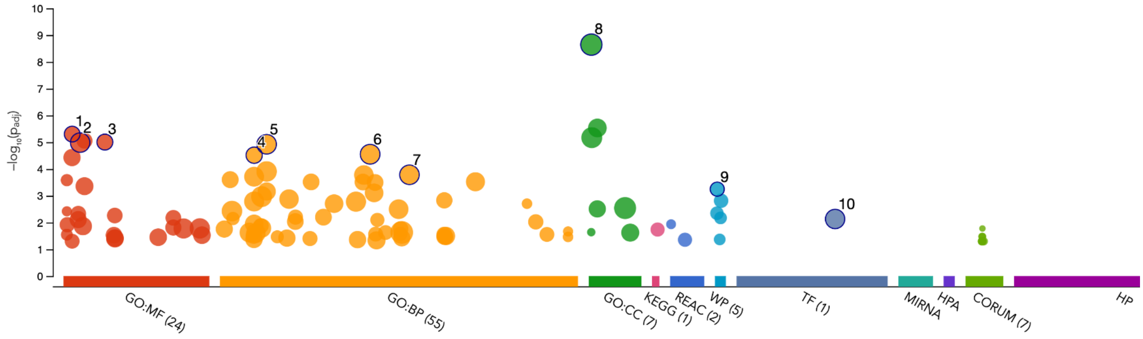

ID	Source	Term ID	Term Name	p_{adj} (query_...
1	GO:MF	GO:0004252	serine-type endopeptidase activity	4.964×10^{-6}
2	GO:MF	GO:0005102	signaling receptor binding	1.033×10^{-5}
3	GO:MF	GO:0017171	serine hydrolase activity	9.877×10^{-6}
4	GO:BP	GO:0006959	humoral immune response	3.051×10^{-5}
5	GO:BP	GO:0009607	response to biotic stimulus	1.191×10^{-5}
6	GO:BP	GO:0044419	biological process involved in interspecies interaction between organisms	2.805×10^{-5}
7	GO:BP	GO:0051707	response to other organism	1.642×10^{-4}
8	GO:CC	GO:0005576	extracellular region	2.261×10^{-9}
9	WP	WP:WP5218	Extrafollicular and follicular B cell activation by SARS-CoV-2	5.656×10^{-4}
10	TF	TF:M07429_1	Factor: SMAD3; motif: CAGACAS; match class: 1	7.362×10^{-3}

Figure S7 Gene ontology enrichment of EpiScore genes used as model training input. Generated as ordered queries using g:Profiler (Kolberg et al., 2023). GO enrichment plots for (a) EpiScores with positive association with chronological age in saliva (42) and (b) EpiScores with negative association with chronological age in saliva (21). Legends show 10 selected GO term names and adjusted p-values for enrichment against all annotated genes.

Supplementary Figure 8

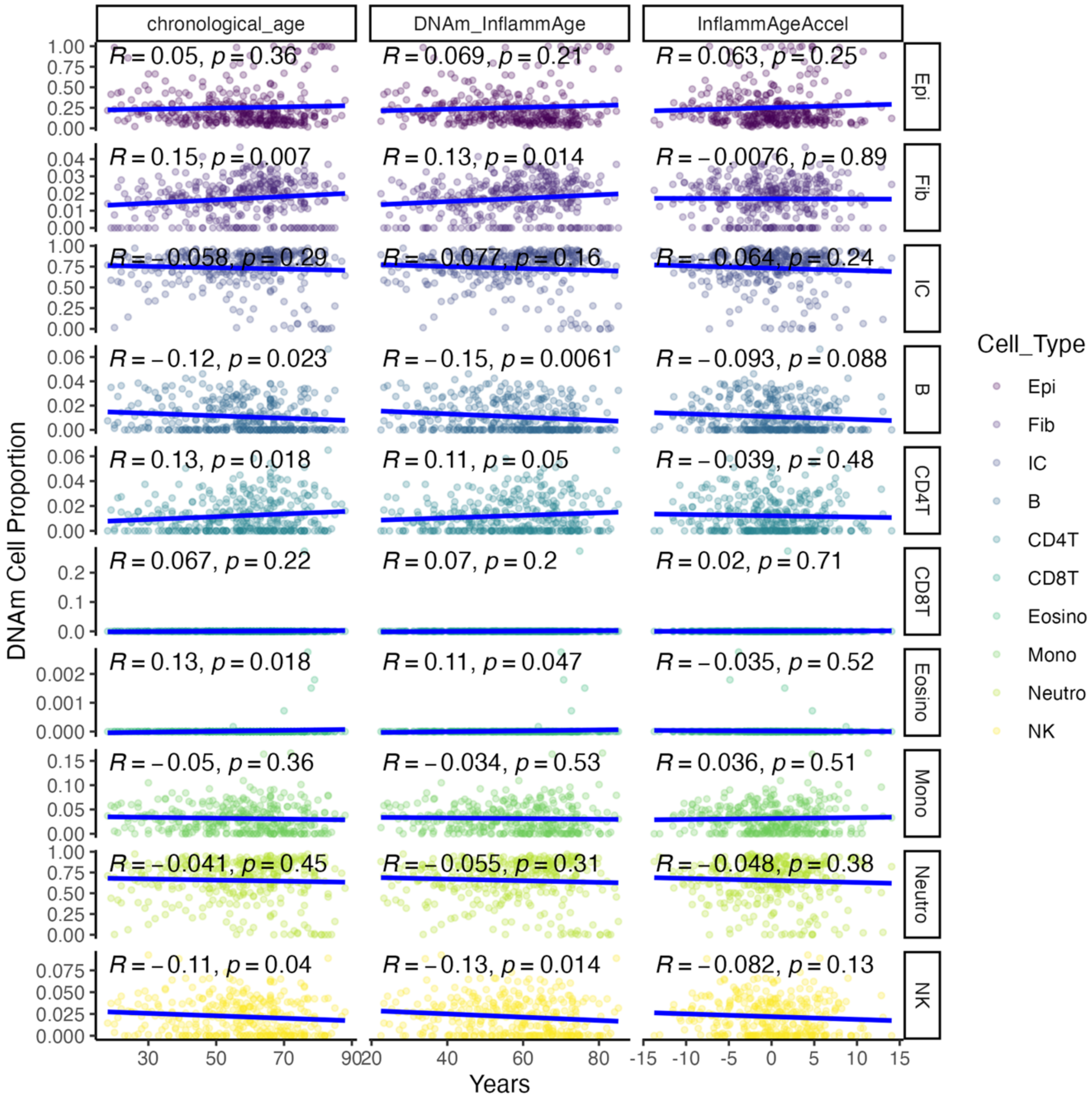

Figure S8 Associations between DNAm-predicted cell type proportions and chronological age (left panels), InflammAge (middle panels) and age-adjusted InflammAge acceleration (InflammAgeAccel, right panels) in the training cohort. Epi: epithelial cells. Fib: fibroblasts. IC: immune cells. B: B cells. CD4T: CD4+ T cells. CD8T: CD8+ T cells. Eosino: eosinophils. Mono: monocytes. Neutro: neutrophils. NK: natural killer cells.

Supplementary Figure 9

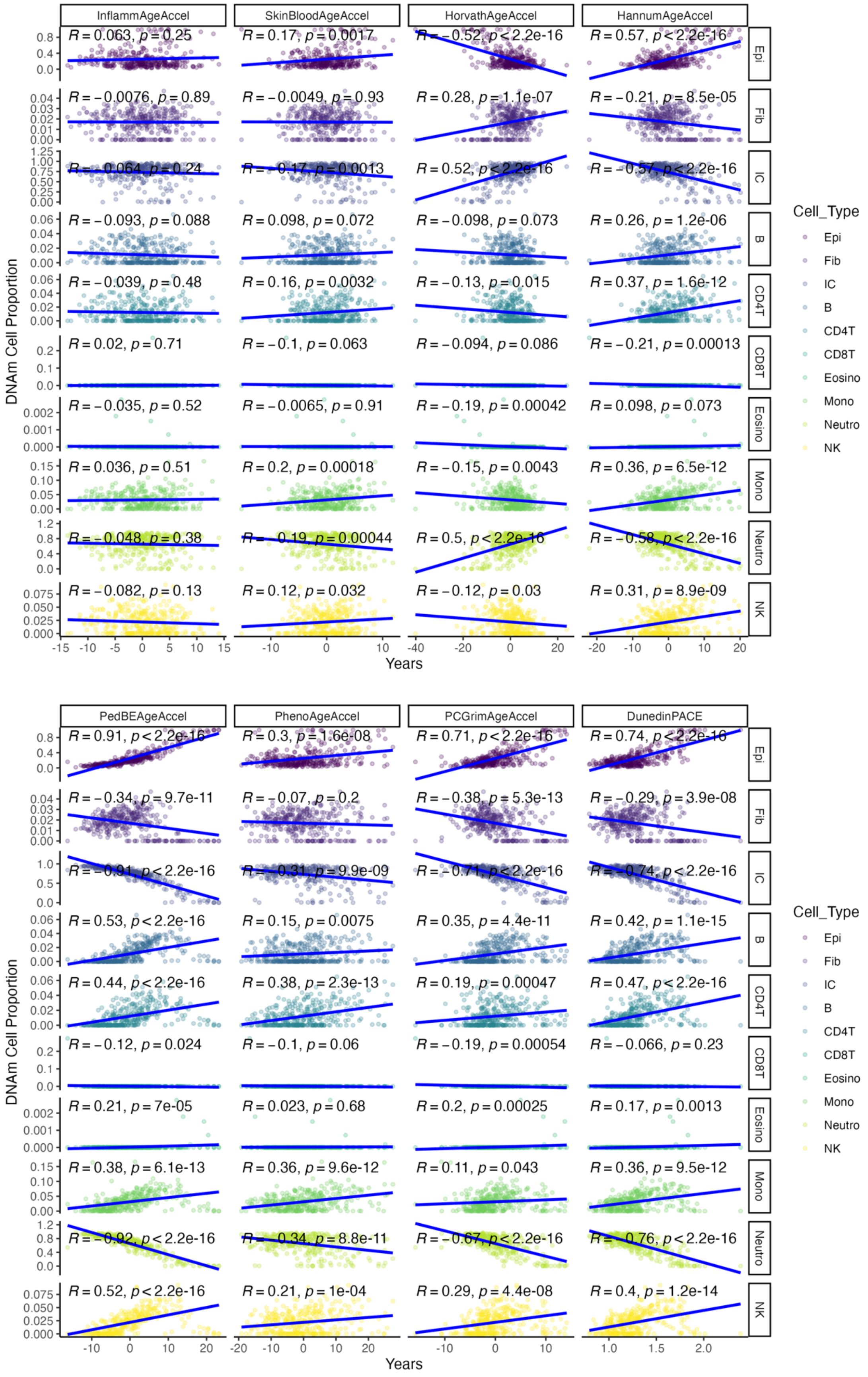

Figure S9 Associations between DNAm-predicted cell type proportions and age-adjusted epigenetic age acceleration for different epigenetic clocks in the training cohort. SkinBlood: skin-blood clock (Horvath et al., 2018). Horvath: multi-tissue Horvath clock (Horvat, 2013). Hannum: Hannum clock (Hannum et al., 2013). PedBE: paediatric clock (McEwen et al., 2019). PhenoAge: principal components PhenoAge clock (Levine et al., 2018). PCGrimAge: principal components GrimAge clock (Higgins-Chen et al., 2022). DunedinPACE: DunedinPACE clock (Belsky et al., 2022). Epi: epithelial cells. Fib: fibroblasts. IC: immune cells. B: B cells. CD4T: CD4+ T cells. CD8T: CD8+ T cells. Eosino: eosinophils. Mono: monocytes. Neutro: neutrophils. NK: NK cells.

Supplementary Figure 10

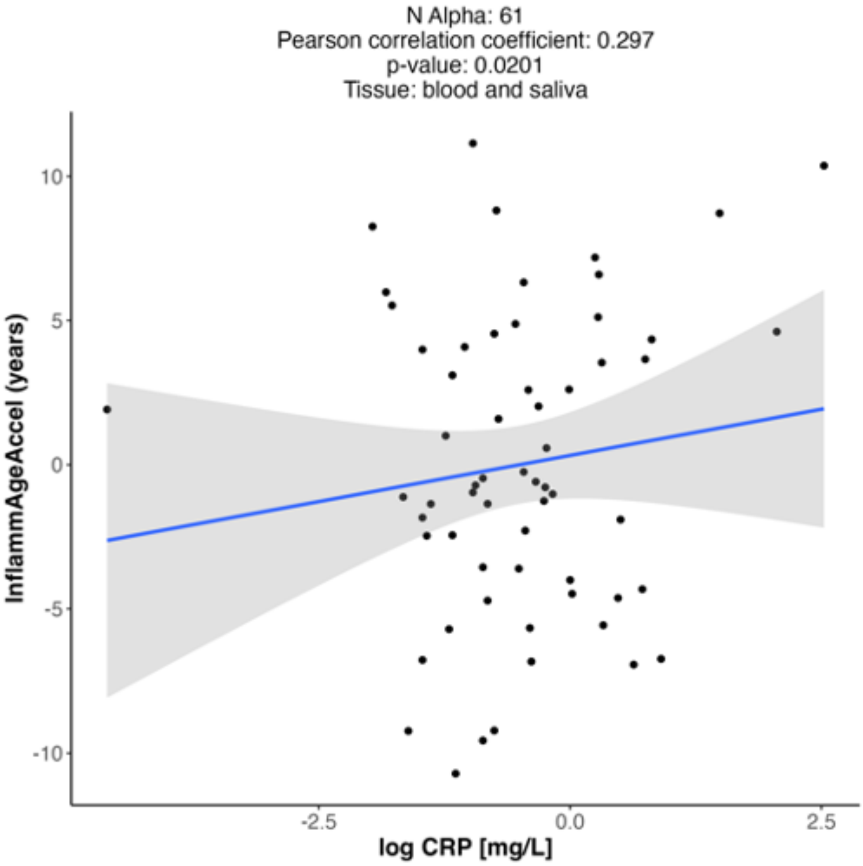

Figure S10 Scatter plot of InflammAge acceleration compared to log-transformed blood CRP in the Alpha test dataset. CRP was measured from blood serum (see **Supp. Methods - Note 1**) and DNAm InflammAge acceleration (InflammAgeAccel) was calculated from saliva DNAm data (N=61).

Supplementary Figure 11

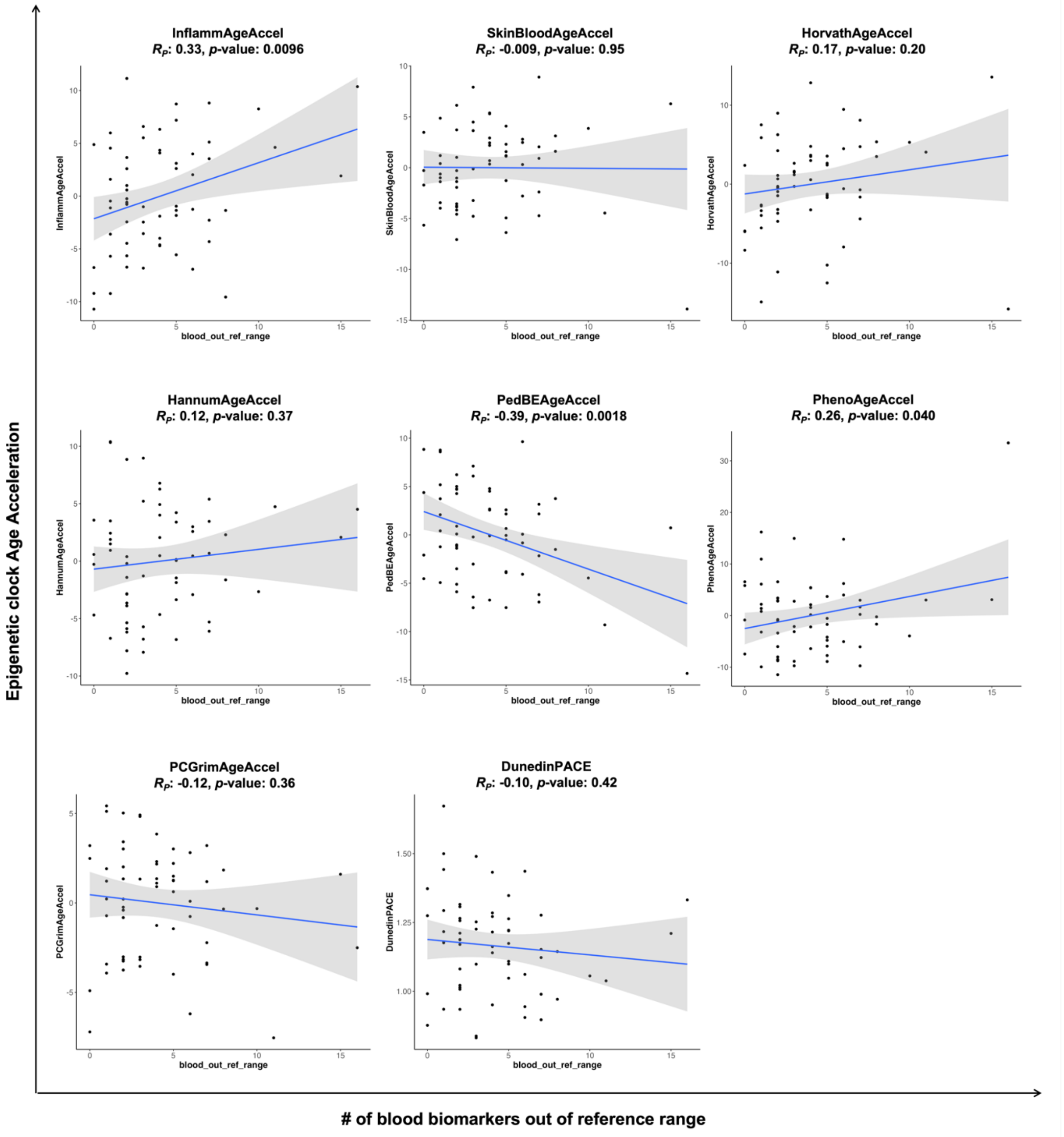

Figure S11 Associations between age-adjusted epigenetic age acceleration (AgeAccel) for different epigenetic clocks and the number of blood SCI biomarkers outside of reference ranges in the Alpha cohort. SkinBlood: skin-blood clock (Horvath et al., 2018). Horvath: multi-tissue Horvath clock (Horvat, 2013). Hannum: Hannum clock (Hannum et al., 2013). PedBE: paediatric clock (McEwen et al., 2019). PhenoAge: principal components PhenoAge clock (Levine et al., 2018). PCGrimAge: principal components GrimAge clock (Higgins-Chen et al., 2022). DunedinPACE: DunedinPACE clock (Belsky et al., 2022).

Supplementary Figure 12

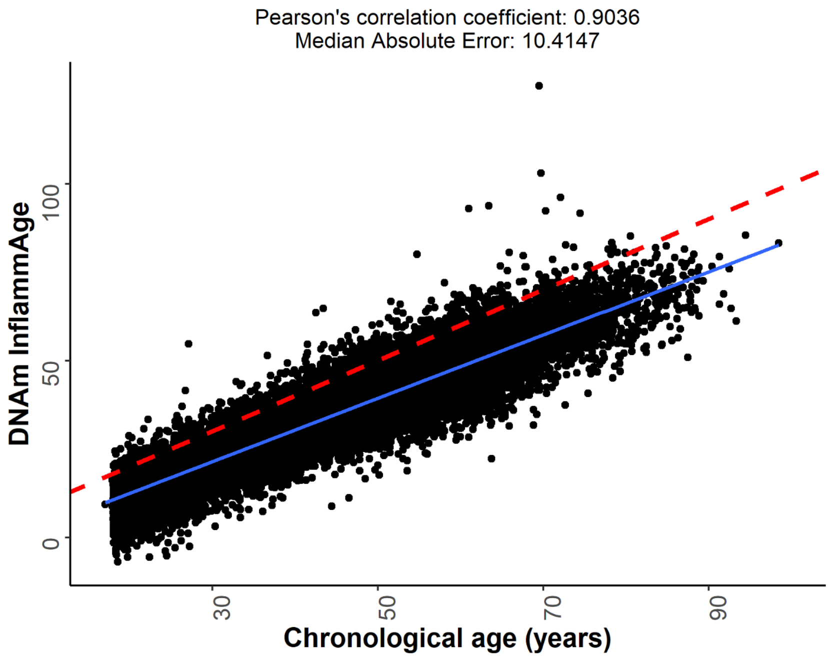

Figure S12 InflammAge performance in GS. InflammAge was calculated for 18,865 participants blood DNAm data and correlated with chronological age. Blue line=linear model fit, red dashed line=diagonal (intercept=0, slope=1).

Supplementary Figure 13

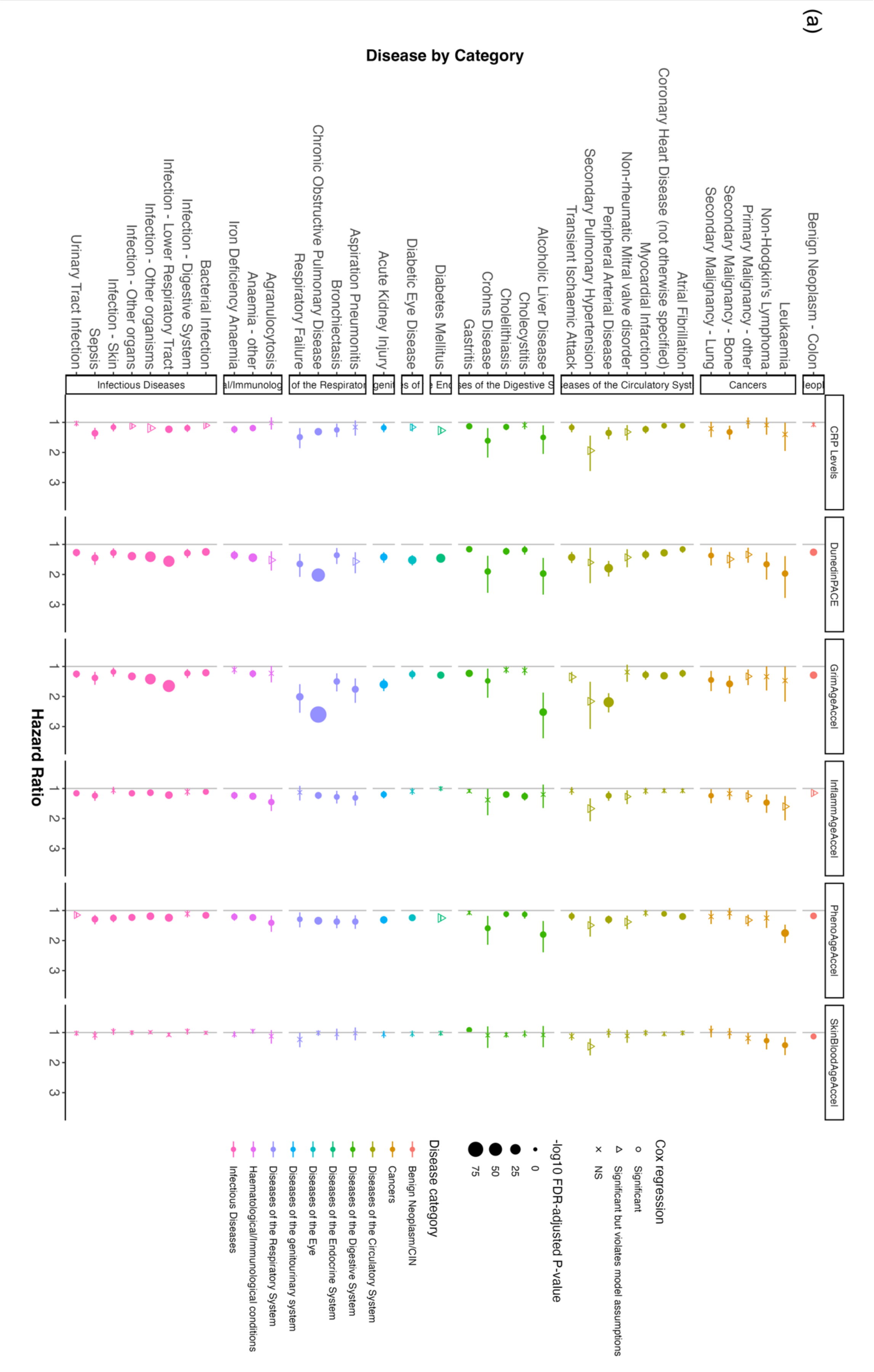

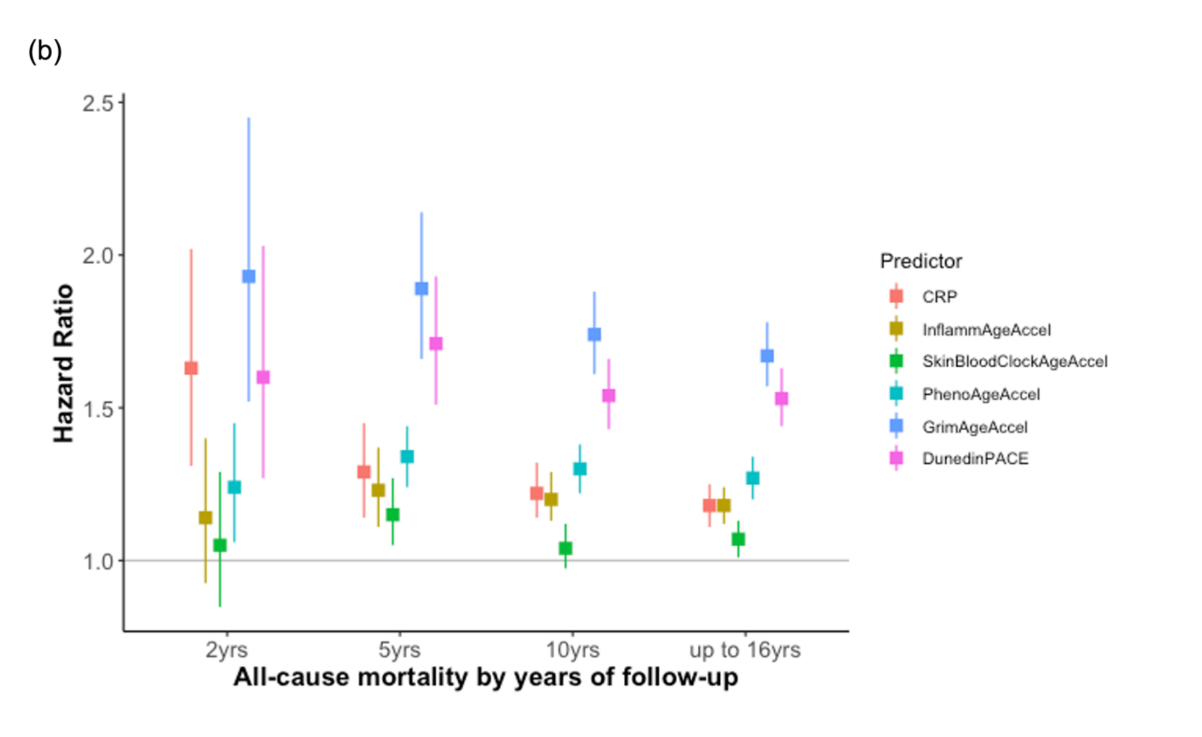

Figure S13 Benchmarking of InflammAge acceleration and clinical endpoints compared to gold standard SCI markers and epigenetic clocks in Generation Scotland. Incidence of clinical endpoint predicted for fully adjusted InflammAge acceleration (InflammAgeAccel), skin-blood clock age acceleration (SkinBloodAgeAccel) and CRP, PhenoAge (PhenoAgeAccel), GrimAge (GrimAgeAccel) and DunedinPACE using covariate-adjusted Cox regression models. (a) Disease incidence Cox regression analysis. Displayed are the same 36 diseases with >30 cases in GS and association FDR-adj. p-value<0.05 in InflammAgeAccel, SkinBloodAgeAccel or CRP. Hazard ratio (HR) and 95% confidence intervals (CI) for all clocks per disease are given in **Table S7**. The plot shows HR and 95% CI across six predictors. Colour=disease category (Kuan et al., 2023), shape=significance (NS=non-significant), filled=no model assumption violations. Point size is proportional to -log10 of FDR-adj. p-value. (b) Mortality prediction in GS for 2 (N=66 events), 5 (N=241 events), 10 years (N=683 events) and the entire follow-up period (N=1,031 events). Squares=HR, lines=95% CI.

Supplementary Figure 14

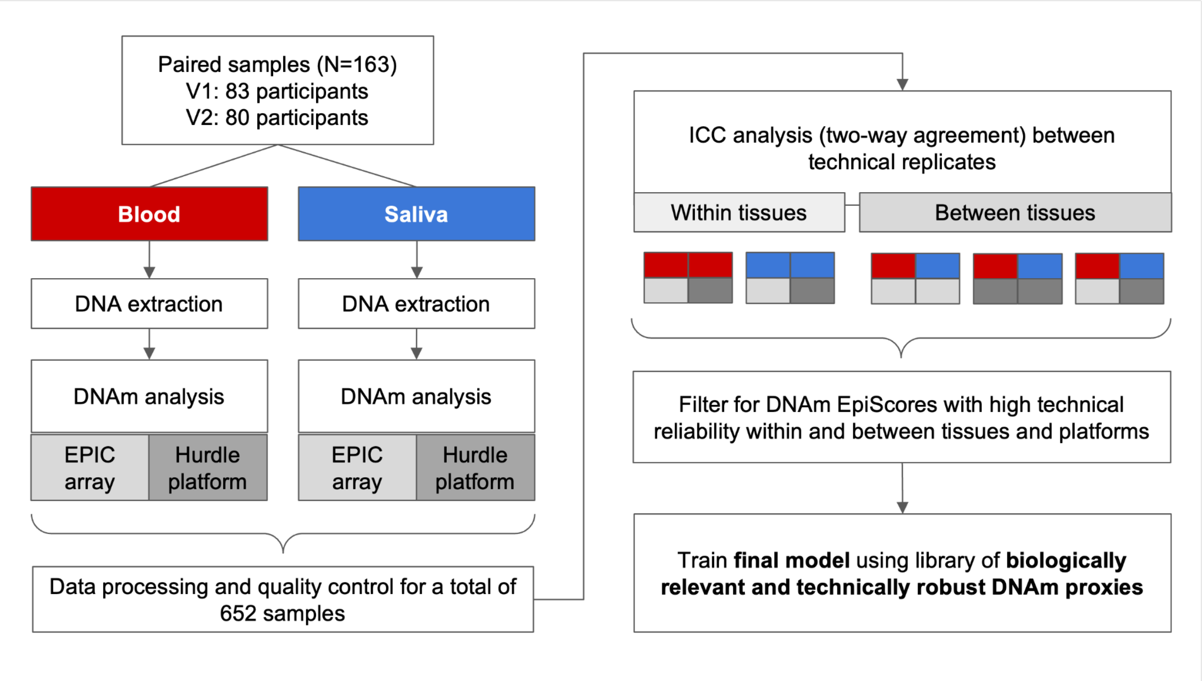

Figure S14 Reliability dataset ICC study design. 83 participants were recruited and DNAm measured in blood and saliva at baseline (V1, N=83) and after 3 months (V2, N=80) and processed in duplicate on the EPIC v1.0 array and the Hurdle DNAm platform. ICC was calculated for the five comparisons indicated. ICCs calculated in V1 samples were used for filtering steps 3-4 of the framework. ICCs calculated in V2 samples were used to assess reliability of the final InflammAge model trained in the training cohort.

Supplementary Tables

Table S1

Table S1 Overview of phenotypes covered on the Hurdle DNAm platform.

Main Phenotype	Example sub phenotypes
Allergy and intolerance	Asthma, cow milk allergy
Autoimmune disease	Crohn’s disease, Rheumatoid arthritis
Biological age	Sex-specific ageing, Ageing, see Table S2 for ageing clocks
Breastfeeding	Breast feeding
Cancer	Breast cancer, lung cancer, melanoma
Cardiovascular risk	Blood pressure, ischemic stroke
Cell composition	EpiScore cell composition
Complex traits	Alcohol, smoking, BMI
Developmental disorder	Cleft palate, cleft lip
Diet	Mediterranean diet, folate, vitamin B12
Ethnicity	Ethnicity African European
Exercise	Exercise
Exposure	Alcohol, smoking, air pollution
Fertility	Assisted Reproductive Technology
Gut	Inflammatory bowel disease, Crohn's disease, ulcerative colitis
Infant	Perinatally acquired HIV, cry acoustics factor 2, neonatal morbidity risk score
Infection	Covid-19, HIV
Inflammageing	Probes associates with inflammation biomarkers in the Alpha dataset
Inflammation	CRP, IL-6, circulating protein proxies
Kidney function	Estimated glomerular filtration rate (eGFR), kidney function, chronic kidney disease
Liver function	Hepatic fat
Lung function	FEV1/FVC, lung cancer

Mental state/disorder	Depression, child abuse, aggressive behavior
Metabolic status	Weight loss, type 1 diabetes, type 2 diabetes, body mass index
Mortality	Mortality, life expectancy
Neurological disorder	Alzheimer's disease, Parkinson's disease
Pregnancy	Maternal body mass index, pre-pregnancy obesity
Protein/metabolite proxies	Circulating protein proxies (including IL-6, CRP, B2M,...)
Sex	Sex-specific ageing, sex, gender
Sleep	Insufficient sleep, sleep narcolepsy

Table S2

Table S2. Summary of EpiScores, epigenetic ageing clocks and other DNAm-based predictors included in the Hurdle DNAm platform design. Probes not on EPIC were not included.

EpiScore name	Type	Publication	Tissue training dataset
Pan-tissue Horvath clock	ageing clock	Horvath 2013	51 healthy tissues and cell types
ELOVL2, FHL2	EWAS age	Garagnani 2012	whole blood
ELOVL2, FHL2	ageing clock	Jung 2019	whole blood + saliva + buccal swab (100 each)
Hannum clock	ageing clock	Hannum 2013	whole blood
DNAm PhenoAge	ageing clock	Levine 2018	whole blood
GrimAge*	ageing clock	Lu 2019a	whole blood
Skin/blood clock	ageing clock	Horvath 2018	whole blood, buccal, skin, epithelium, fibroblast, cord blood
Telomere length clock	ageing clock	Lu 2019b	whole blood
DunedinPoAm (Pace of Aging)	ageing clock	Belsky 2020	whole blood
DunedinPACE*	ageing clock	Belsky 2022	whole blood
CellAge clock	ageing clock	Lujan 2020	human mammary fibroblasts + human dermal fibroblasts
Pediatric-Buccal-Epigenetic PedBE clock	ageing clock	McEwen 2020	paediatric buccal swabs
epiTOC2 mitotic clock	ageing clock	Teschendorff 2020	whole blood
Cancer clock	ageing clock	Zhu 2019	33 cancer types + matched normal tissue (trained in normal tissue control samples and tested in 7,988 tumour samples)
MRScore, mortality	EWAS and mortality risk score	Zhang 2017	whole blood
Mortality 2	EWAS meta-analysis + mortality risk score	Colicino 2020	whole blood
Life expectancy	ageing clock	Lin 2016	whole blood
Skin age**	ageing clock	Boroni 2020	human skin biopsies (40 derived from the dermis, 146 from the epidermis, and 322 samples derived from whole skin tissue)

EpiScore name	Type	Publication	Tissue training dataset
Brain clock	ageing clock	Shireby 2020	cortex
Gestational Age	EWAS meta-analysis	Merid 2020	cord blood and whole blood
SmPEGS	smoke predictor	Sudgen 2019	whole blood
CRP epi signature	inflammatory protein EWAS	Ligthart 2016	whole blood
CRP epi signature 2	inflammatory protein predictor	Stevenson 2020	whole blood
IL-6 epi signature	inflammatory protein predictor	Stevenson 2021	whole blood
TNF alpha signature	inflammatory protein EWAS	Aslibekyan 2018	whole blood
i-ePGS (inflammation-related epigenetic polygenic risk score)	inflammatory protein predictor	Barker 2018	cord blood and whole blood
Inflammatory proteins	inflammatory protein predictor	Hillary 2020	whole blood
obesity SOCS3	EWAS	Ali 2016	peripheral blood mononuclear cells
Type 2 diabetes 10-year	Metabolic status	Cheng 2021	whole blood
Major Depressive Disorder	MDD risk score	Barbu 2021	whole blood
SOMAscan and Olink-based EpiScores	Circulating plasma protein predictors (109)	Gadd 2022	whole blood
Circulating plasma protein pQTMs	pEWAS of plasma proteins	Zaghlool 2020	whole blood
Serum urate	EWAS	Tin 2021	whole blood
Neurological proteins	pEWAS of neurological proteins	Hillary 2019	whole blood
BMI	complex trait predictor	McCartney 2018	whole blood
Type 1 Diabetes longitudinal	case-control EWAS	Johnson 2020	whole blood

*Some predictors require data normalisation with additional sites, not all of which could be included in the Hurdle DNAm platform due to limitations in the maximum number of probes allowed in the array.
**only 50 top CpG sites published

Table S3

Table S3 Overview of cohorts included in the InflammAge training dataset.

Cohort	Platform	Tissue	Ethnicity	Disease status
E-MTAB-5377	Illumina Infinium Methylation450 BeadChip	Saliva	British	Control
GSE92767, GSE59505, GSE59508		Saliva	Korean-Asian	Control
GSE111223-GSE78874		Saliva	European + Hispanic	Control
GSE99029		Saliva	South African Khomani San	Control
Hurdle in-house dataset	Illumina Infinium MethylationEPIC v1.0 BeadChip	Saliva	Not reported	Not reported

Table S4 - Legend

Table S4 Pearson correlations between chronological age or number of blood SCI biomarkers outside the reference range (see Figure S10) correlated with epigenetic clock values or respective age-adjusted age acceleration in the Alpha cohort. SkinBlood: skin-blood clock (Horvath et al., 2018). Horvath: multi-tissue Horvath clock (Horvat, 2013). Hannum: Hannum clock (Hannum et al., 2013). PedBE: paediatric clock (McEwen et al., 2019). PhenoAge: principal components PhenoAge clock (Levine et al., 2018). PCGrimAge: principal components GrimAge clock (Higgins-Chen et al., 2022). DunedinPACE: DunedinPACE clock (Belsky et al., 2022), chronoage: chronological age, blood_out_of_ref_range: number of blood SCI biomarkers outside the reference range, AgeAccel: epigenetic age acceleration, R_p: Pearson correlation coefficient.

[Schmunk_et_al_2024_SupplementaryTables.xlsx]

Table S5

Table S5 Summary information for the GS test cohort. It includes information for the risk factors that were included in the Cox regression models.

**Total cohort size**	N = 18865
**InflammAgeAccel**	
Mean (SD)	-0.000322 (6.32)
Median [Min, Max]	-0.106 [-29.5, 70.9]
**Age**	
Mean (SD)	47.6 (14.9)
Median [Min, Max]	49.2 [17.1, 98.5]
**Sex**	
Male	7771 (41.2%)
Female	11094 (58.8%)
**Smoking Status**	
Non-smoker	5402 (28.6%)
Ex-smoker	9636 (51.1%)
Smoker	3228 (17.1%)
Missing	599 (3.2%)
**Socioeconomic status (SIMD)**	
Mean (SD)	3900 (1850)
Median [Min, Max]	4340 [1.00, 6510]
Missing	1153 (6.1%)
**Years of Education**	
0	7.00 (0.0%)
1-4	50.0 (0.3%)
5-9	546 (2.9%)
10-11	4954 (26.3%)
12-13	3888 (20.6%)
14-15	2563 (13.6%)
16-17	3525 (18.7%)
18-19	1699 (9.0%)
20-21	431 (2.3%)
22-23	128 (0.7%)
24	62.0 (0.3%)
Missing	1012 (5.4%)
**Body Mass Index (kg/m2)**	

Mean (SD)	26.7 (5.18)
Median [Min, Max]	25.9 [10.5, 71.4]
Missing	120 (0.6%)
**Pack Years**	
Mean (SD)	8.30 (16.4)
Median [Min, Max]	0 [0, 216]
Missing	395 (2.1%)

Table S6

Table S6 Spearman correlation coefficients between InflammAgeAccel and basic demographic and biochemistry variables in GS (N=18,865).

Trait	Spearman correlation coefficient R_S
HDL cholesterol	-0.06
Total cholesterol	-0.06
Deprivation*	-0.06
Education	-0.04
Sodium	-0.04
Urea	-0.03
Age	-0.01
Troponin I	-0.01
Potassium	0
Ankle brachial pressure index	0
% body fat	0.01
Alcohol	0.01
BMI	0.02
Glucose	0.02
Creatinine	0.03
Troponin T	0.03
Waist-hip ratio	0.04
NTproBNP	0.05
GDF15	0.06
Smoking pack years	0.08
CRP	0.11
DNAm inflammage	0.39

*coded such that high values indicate less deprivation

Table S7 - Legend

Table S7 Cox regression summary statistics across 36 diseases with >30 cases and significant association in at least one of 3 predictors (CRP, InflammAgeAccel, SkinBloodAgeAccel) in GS, including results for other gold standard epigenetic clocks (PhenoAgeAccel, GrimAgeAccel, DunedinPACE). The table is sorted by decreasing hazard ratio (HR) per disease.

[Schmunk_et_al_2024_SupplementaryTables.xlsx]

Table S8 - Legend

Table S8 Summary statistics across dietary lifestyle factors and InflammAgeAccel in GS. Tukey summary statistics for an association between InflammAgeAccel and different dietary variables collected at baseline during GS participant recruitment. **‘**diff’: mean difference in InflammAgeAccel between groups. ‘lwr’: lower 95% confidence interval. ‘upr’: upper 95% confidence interval. ‘p.adj’: Tukey p-value adjusted for multiple comparisons.

[Schmunk_et_al_2024_SupplementaryTables.xlsx]
